# Compound heterozygous *SLC12A5* variants expand the molecular and functional spectrum of KCC2‐developmental and epileptic encephalopathy

**DOI:** 10.1002/epi.70258

**Published:** 2026-04-25

**Authors:** Mira Hamze, Robyn Whitney, Dorothée Ville, Nathalie Villeneuve, Anna‐Maria Hartmann, Lisa Becker, Jens Hausmann, Jinwei Zhang, Cathy Brier, Lucie I. Pisella, Perrine Friedel, Audrey Labalme, Eudeline Alix, Nicolas Chatron, Damien Sanlaville, Sylvie Gory‐Fauré, Eric Denarier, Christophe Porcher, Gaetan Lesca, Igor Medina

**Affiliations:** ^1^ Aix‐Marseille University, Institut de Neurobiologie de la Méditerranée, Institut National de la Santé et de la Recherche Médicale Marseille France; ^2^ Division of Neurology, Department of Pediatrics McMaster University Hamilton Ontario Canada; ^3^ Department of Pediatric Neurology and Reference Center of Rare Epilepsies, University Hospitals of Lyon Bron France; ^4^ Department of Pediatric Neurology Assistance Publique–Hôpitaux de Marseille, La Timone Children's Hospital Marseille France; ^5^ Division of Neurogenetics, Faculty VI, School of Medicine and Health Sciences Carl von Ossietzky University Oldenburg Oldenburg Germany; ^6^ Research Center for Neurosensory Sciences Carl von Ossietzky University Oldenburg Oldenburg Germany; ^7^ Division of Anatomy, School of Medicine and Health Sciences Carl von Ossietzky University Oldenburg Oldenburg Germany; ^8^ State Key Laboratory of Chemical Biology, Research Center of Chemical Kinomics Shanghai Institute of Organic Chemistry, Chinese Academy of Sciences Shanghai China; ^9^ Department of Medical Genetics, member of European Reference Network EpiCARE, University Hospitals of Lyon University Claude Bernard Lyon Lyon France; ^10^ Pathophysiology and Genetics of Neuron and Muscle NeuroMyoGene Institute—Centre National de la Recherche Scientifique UMR5261—Institut de Neurobiologie de la Méditerranée U1315, Lyon 1 Université Claude Bernard Lyon France; ^11^ Université Grenoble Alpes, Institut de Neurobiologie de la Méditerranée, U1216, CEA, Grenoble Institut des Neurosciences Grenoble France

**Keywords:** chloride, GABA, KCC2, neuron, *SLC12A5*

## Abstract

**Objective:**

This study was undertaken to characterize the functional impact of novel *SLC12A5* variants in two unrelated patients with early onset developmental and epileptic encephalopathy (DEE) and to investigate the mechanisms underlying KCC2 dysfunction.

**Methods:**

Clinical, genetic, and functional analyses were performed in two patients (Cases A and B) with DEE. *SLC12A5* encodes two KCC2 splice isoforms (KCC2a and KCC2b). Functional effects of the identified variants on KCC2b ion transport, phosphorylation, mRNA processing, and KCC2‐dependent synaptogenesis were assessed using in vitro assays in heterologous expression systems and primary neurons, supported by in silico structural modeling.

**Results:**

Both patients developed severe neonatal onset DEE characterized by developmental delay, axial hypotonia, extrapyramidal features, and bilateral migratory seizures within 24 h of birth. Both cases resulted in early mortality (Case A at 9 years; Case B at 6 months). Sequencing revealed distinct biallelic compound heterozygous *SLC12A5* variants in both individuals, each inherited from one unaffected parent. Functional analyses demonstrated that in Case A, one variant markedly reduced KCC2‐mediated ion transport, whereas the second variant preserved transport activity but exhibited an altered phosphorylation profile at Ser940, located on the intracellular *C*‐terminal region. This variant also disrupted wild‐type (WT) KCC2‐dependent excitatory synapse formation in immature rat hippocampal neurons. In Case B, one variant disrupted normal mRNA transcript processing consistent with loss of expression, and the second variant exhibited reduced ion transport activity.

**Significance:**

These data demonstrate that *SLC12A5*‐related DEE can result from combined impairment of KCC2‐dependent chloride homeostasis and disruption of chloride‐independent KCC2 functions critical for early neuronal development. This work expands the mutational and mechanistic spectrum of *SLC12A5*‐DEE and highlights the importance of KCC2 regulatory roles in early brain development, providing new knowledge and tools for basic research and potential avenues for targeted precision therapies.

## Graphical abstract

Overview of the multimodal experimental approach integrating clinical, genetic, in silico, and in vitro investigations. Clinical: Representative EEG recording setup and ictal traces from affected patients. Genetic: Pedigrees for Families A and B highlighting the inheritance of the four identified *SLC12A5* variants (A1, A2, B1, B2). In silico: 3D structural model of KCC2 illustrating the spatial localization of the substituted amino acids. In vitro: Schematic representations of mRNA processing and translation, KCC2‐mediated ion transport, and the impact of variants on hippocampal pyramidal neurons.

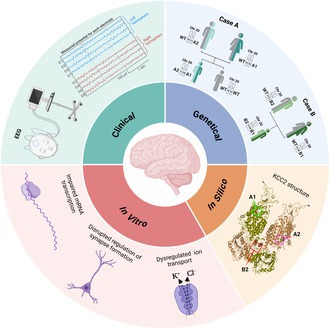

**Key points**

- Biallelic variants in *SLC12A5* cause a severe neonatal‐onset developmental and epileptic encephalopathy.
- Pathogenic mechanisms include impaired ion transport, altered KCC2 structure and regulation, and defective mRNA processing.
- A variant preserving ion transport disrupts KCC2‐dependent synaptogenesis, supporting chloride‐independent disease mechanisms.
- These findings expand the mechanistic spectrum of *SLC12A5*‐related encephalopathy beyond canonical loss of KCC2 ion‐transport function.

## 1 INTRODUCTION

Developmental and epileptic encephalopathies (DEEs) are a group of devastating conditions characterized by frequent refractory seizures, profound psychomotor delay, and other neurological features, caused by pathogenic variants in a growing number of genes.

The *SLC12A5* gene encodes the neuronal potassium‐chloride cotransporter KCC2, a 12‐transmembrane domain (TD) protein essential for extruding intracellular chloride and maintaining γ‐aminobutyric acidergic (GABAergic) and glycinergic inhibition.^1^, ^2^ Beyond its canonical role in ion transport, KCC2 is also involved in noncanonical, ion transport‐independent processes related to synaptic development and neuronal maturation.^3^

Although studies in animal models and human brain tissue had long implicated KCC2 in epilepsy and neurodevelopmental disorders, the identification of pathogenic *SLC12A5* variants in patients represented a major breakthrough in understanding its role in disease. The first group of disease‐associated variants included at least three different heterozygous autosomal dominant missense *SLC12A5* variants identified in individuals with relatively mild epilepsy phenotypes, including febrile seizures^4^ and idiopathic generalized epilepsy.^5^, ^6^ However, these same variants have also been found in unaffected individuals, suggesting incomplete penetrance and supporting a model in which one functional allele suffices to preserve KCC2 function.

A major advance came with the discovery of patients harboring recessive biallelic pathogenic *SLC12A5* variants.^7^, ^8^, ^9^, ^10^ The nine cases reported so far were consistently associated with severe DEE, most notably epilepsy of infancy with migrating focal seizures (EIMFS; Table S1). Functional studies confirmed that these variants impair KCC2‐mediated chloride extrusion, leading to disrupted inhibitory signaling during early brain development. Structural modeling predicted deleterious effects of these variants on KCC2 stability, and in vitro assays revealed glycosylation, reduced membrane expression, and diminished ion transport activity.^7^, ^8^, ^10^ Collectively, these findings supported a disease model in which KCC2 dysfunction disrupts chloride homeostasis, contributing to GABA‐mediated network hyperexcitability.

**FIGURE 1:**
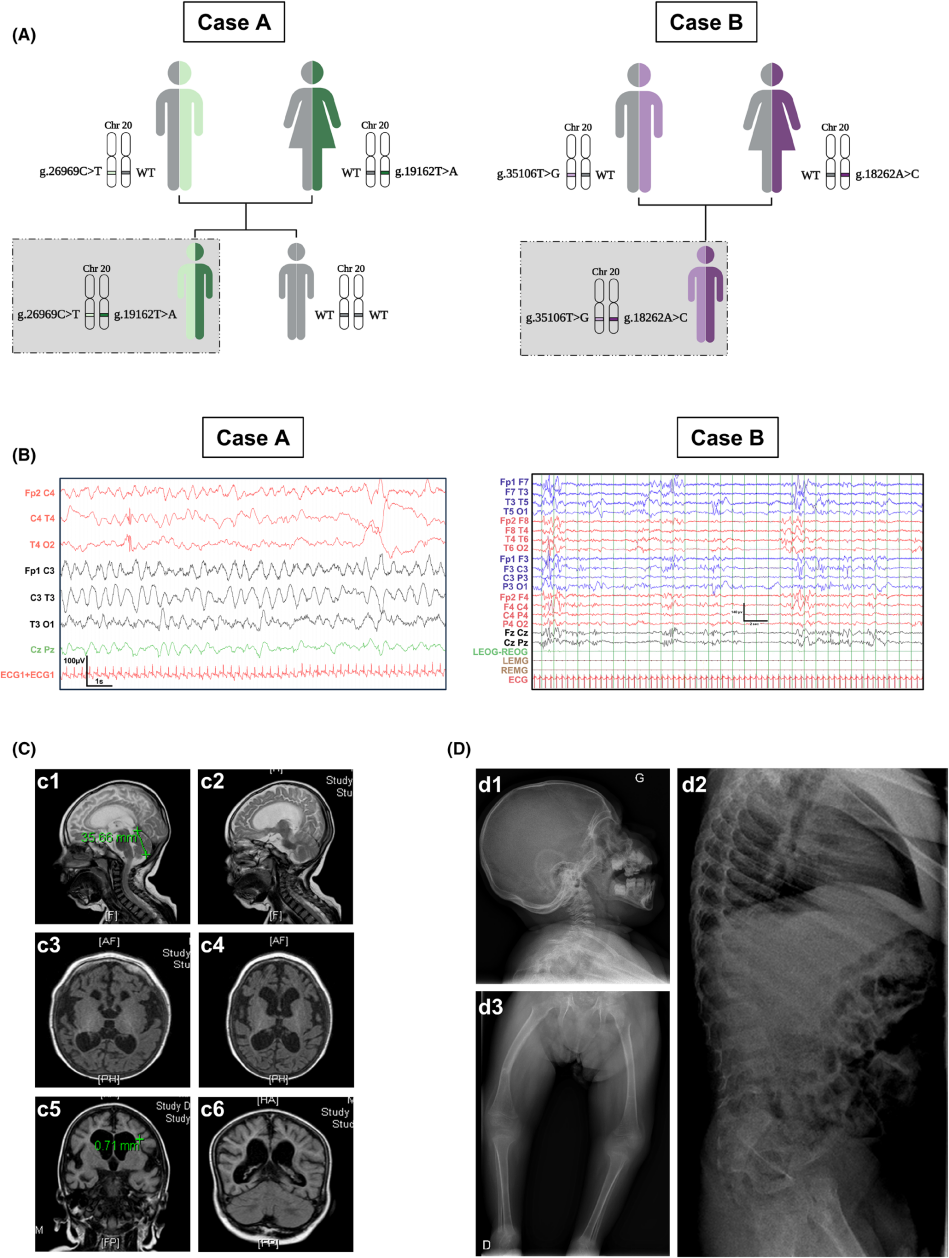
Evidence on genetic disease in Families A and B. (A) Pedigrees of Families A (left panel) and B (right panel) illustrating inheritance of the genetic disorder. Affected individuals are indicated by shaded rectangles. WT denotes the wild‐type allele on chromosome 20. The pathogenic variants are denoted g.19162T>A and g.26969C>T for Case A and g.18262A>C and c.2366T>G for Case B (here and throughout, variant numbering refers to the genomic reference sequence NG_046341.2 [hg19]). (B) Representative electroencephalographic (EEG) recordings from patient A (left panel) and patient B (right panel), obtained at 6 months and 5 days of age, respectively. Recordings show a discontinuous background with multifocal epileptiform discharges. Additional EEG examples are provided in Figures S1 and S2. (C) Brain magnetic resonance imaging of Patient A at 2 years of age demonstrating abnormalities on sagittal T2‐weighted (c1), axial T1‐weighted (c2), and coronal T2‐weighted (c3) images. (D) Skeletal radiographs of Patient A obtained at 4 years of age, including the skull (d1), chest (d2), and lower body (d3).

Here, we describe two new cases of compound heterozygous *SLC12A5* variants associated with a severe form of DEE with drug‐resistant EIMFS presenting within hours of birth (Figure 1A,B). Functional in vitro and in silico analyses revealed molecular defects distinct from previously reported variants, suggesting additional pathogenic mechanisms beyond canonical loss of function that contribute to KCC2‐DEE.

## 2 MATERIALS AND METHODS

A detailed description of the methods is available in the Supporting Information. Most procedures have been described in prior publications, which are cited in the relevant Results sections. Written informed consent was obtained from the caregivers of the patients in accordance with local university/hospital ethics procedures.

Statistical analyses and normality assessments (Shapiro–Wilk test) were performed using Prism 10 (GraphPad). All datasets either failed the normality test or included small sample sizes (*n* < 20); therefore, nonparametric tests indicated in figure legends were used to compare independent groups. The details of the statistical tests are provided in the Supporting Information. Values reported in the text represent mean differences or are presented as mean ± SD.

## 3 RESULTS

### 3.1 Clinical features

Case A was a boy born at term after an uneventful pregnancy to healthy, nonconsanguineous parents. Delivery was vaginal at 39 weeks of gestation, with normal birth parameters and Apgar scores of 10/10. Seizures began at 3 h of life and rapidly became frequent, occurring daily without seizure‐free intervals. Seizures were predominantly focal with a tonic component, often occurring in clusters, and ranged from several per day to more than 100 events daily. No myoclonic seizures, epileptic spasms, or generalized tonic–clonic seizures were observed.

Early electroencephalographic (EEG) recordings demonstrated multiple multifocal electroclinical seizures with a focal migrating pattern (Figures 1B and S1). Serial EEGs throughout childhood consistently showed severely abnormal background activity with multifocal epileptiform discharges, predominantly over the frontocentral regions. Over time, progressive background slowing, poor organization, and recurrent focal or multifocal paroxysmal discharges were observed. Several recordings captured tonic seizures, including reflex seizures triggered by auditory stimuli.

Brain magnetic resonance imaging (MRI) was normal at 3 months of age but revealed diffuse cerebral atrophy by 2 years, consistent with progressive structural involvement (Figure 1C).

Neurologically, the child exhibited profound developmental impairment from infancy. He never achieved independent sitting, purposeful grasping, or consistent visual tracking, and showed minimal spontaneous movements outside seizure episodes. Muscle tone was characterized by a persistent generalized hypertonic posture that did not clearly correspond to a classic pyramidal or extrapyramidal syndrome. Moderate respiratory fragility was present from early infancy. Growth parameters progressively declined, with weight falling below the 3rd centile and head circumference below the 1st centile. He also experienced recurrent bone fractures, suggesting increased bone fragility.

Seizures remained drug resistant despite trials of multiple antiseizure medications (ASMs), vagus nerve stimulation, and the ketogenic diet (see Supporting Information). Over time, his neurological condition progressively deteriorated, with worsening hypertonia, loss of feeding autonomy requiring gastrostomy placement, and recurrent hospitalizations for seizure exacerbations and respiratory distress. He died at 9 years of age following a decision to limit acute care in the context of severe neurological disease.

Case B was a boy born at 38 weeks of gestation by cesarean section because of a nonreassuring biophysical profile. Family history was noncontributory. At birth, neurological examination revealed increased muscle tone and a poor gag reflex. Seizures began at 6 h of life and were characterized by asymmetric tonic stiffening associated with apnea, oxygen desaturation, tachycardia, and loud crying. Continuous EEG monitoring initiated on day 4 of life demonstrated frequent electrographic and electroclinical tonic seizures arising from multiple brain regions (Figures 1B and S2). The EEG background was markedly discontinuous and associated with multifocal epileptiform discharges. A follow‐up EEG at 2 months showed persistent multifocal epileptiform abnormalities and brief electrographic seizures. Between 2 and 6 months of age, seizures occurred daily, often in clusters.

Brain MRI performed on day 5 of life was normal. Extensive metabolic and infectious investigations were negative. Despite trials of multiple ASMs, seizures remained poorly controlled; partial reduction was achieved after initiation of a classic ketogenic diet (see Supporting Information).

Development was profoundly impaired. The child demonstrated some periods of wakefulness, spontaneous eye opening, and inconsistent responses to sensory stimuli. At 6 months, neurological examination showed marked axial and appendicular hypotonia and absence of consistent visual tracking. He died at 6 months of age due to complications related to his severe neurological condition.

### 3.2 Genetic findings

#### 3.2.1 Case A

**FIGURE 2:**
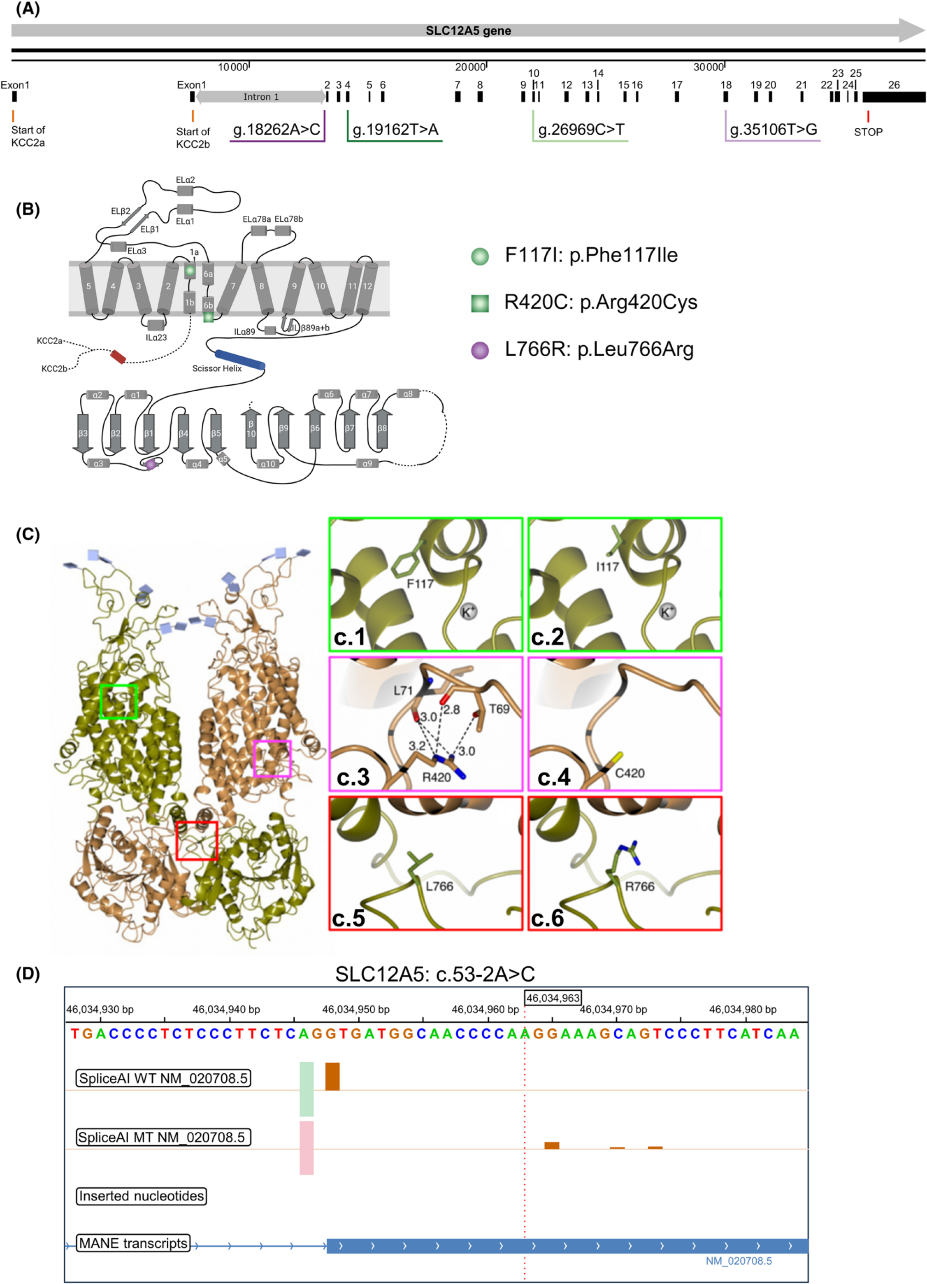
Position and effects of variants in Cases A and B at the genetic, protein, and structural levels. (A) Schematic representation of the full‐length *SLC12A5* gene, composed of 26 exons (black boxes). In Case A, variants g.19162T>A and g.26969C>T are located in exons 4 and 10, respectively. In Case B, variants g.18262A>C and c.2366T>G are located in intron 1 and exon 18, respectively. The *SLC12A5* gene undergoes alternative splicing at exon 1, generating two KCC2 splice variants, KCC2a and KCC2b, which arise from distinct first exons driven by independent promoters. (B) Membrane topology diagram of KCC2 showing distinct and shared regions of the KCC2a and KCC2b splice variants, along with putative transmembrane domains and intra‐/extracellular regions. KCC2a and KCC2b differ in their first 40 and 17 amino acids, respectively, but share 1099 amino acids (aa), including 12 transmembrane domains (TD; 533 aa) and a long intracellular *C*‐terminus (481 aa) that contains many regulatory sites. In the scheme, only variants corresponding to the KCC2b splice variant are shown for simplicity. Variants p.Phe117Ile and p.Arg420Cys are located in the first and sixth TD, respectively. Variant p.Leu766Arg is located in the intracellular *C*‐terminus between the last TD and the region harboring multiple regulatory phosphorylation sites. (C) Structural overview of the KCC2b dimer, colored in olive and brown. Glycans are presented as light blue Glycoblocks.^11^ (c.1, c.3, c.5) Zoom into wild‐type residues Phe117 (F117), Arg420 (R420), and Leu766 (L766). (c.2, c.4, c.6) Substituted residues in patient‐derived variants Phe117Ile (I117), Arg420Cys (C420), and Leu766Arg (R776). Hydrogen bonds are indicated as dashed lines, with atomic distances given in angstroms. A K^+^ ion is shown as a light gray sphere. Structural optimization of R420 has been performed using one round of real space refinement in Coot,^12^ and representations were generated using CCP4mg^13^ (more details in Supporting Information). (D) In silico predictions of exon 2 transcription based on the MANE (Matched Annotation from NCBI and EMBL‐EBI) transcript and a splice variant. Visualization of the effect of the NM_020708.5:c.53‐2A>C variant using SpliceAI‐Visual^14^ indicates complete loss of the physiological splice site (Table S2). A similar prediction of abrogated transcript processing was obtained for another splice variant, NM_001134771.2:c.122‐2A>C (Table S3). To experimentally assess the impact of the c.53‐2A>C variant on *SLC12A5* transcription, we conducted a minigene splicing assay (see Figures S3–S5).

Exome sequencing of Case A and his parents revealed two novel missense variants in *SLC12A5*, which encodes two neuronal splice isoforms, KCC2a and KCC2b (Figures 1A and 2A, Supporting Information). The first *SLC12A5* variant was identified as NG_046341.2:g.19162T>A (hg19), a single‐nucleotide change in exon 10, resulting in transcript‐specific consequences: NM_001134771.2:c.418T>A (p.Phe140Ile) in the KCC2a splice variant and NM_020708.5:c.349T>A (p.Phe117Ile) in the KCC2b splice variant. The second variant was identified at NG_046341.2:g.26969C>T (hg19), resulting in NM_001134771.2:c.1327C>T (p.Arg443Cys) in KCC2a and NM_020708.5:c.1258C>T (p.Arg420Cys) in KCC2b. The first variant was absent from the Genome Aggregation Database (gnomAD v4.1.0) and the All of Us Research Program database, whereas the latter variant was reported once in the heterozygous state. Each variant was inherited from an asymptomatic parent (Figure 2A,B). No other pathogenic/likely pathogenic variants were detected in DEE‐related genes (e.g., *SCN1A*, *KCNQ2*, *CDKL5*) after comprehensive analysis of coding regions, splice sites, and copy number variants.

The position of the first variant fits to the first TD common for both splice variants, whereas the position of the second variant was in the 6th TD (Figure 2B). In silico predictions indicated both variants are highly deleterious (details in Supporting Information). Structural modeling using full‐length human KCC2b (protein database PDB: 6m23)^15^ showed that variant Phe117Ile lies near the bound K^+^ ion, potentially impairing ion transport (Figure 2C, c.1 and c.2, Supporting Information). Variant Arg420Cys disrupts a hydrogen‐bond network, possibly releasing the *N*‐terminus and activating the transporter (Figure 2C, c.3 and c.4).

#### 3.2.2 Case B

An epilepsy gene panel identified two previously unreported *SLC12A5* variants, each inherited from a healthy parent (Figure 2A). Neither variant was present in gnomAD v4.1.0 or AllofUS population databases. One variant affected the canonical splice acceptor site in intron 1 (NG_046341.2:g.18262A>C, hg19). In silico analysis demonstrated that the variant affects a common splice acceptor site used by both the NM_001134771.2 and NM_020708.5 transcripts. SpliceAI predicted near‐complete loss of the canonical acceptor site (ΔAL = .99), with an additional prediction of a cryptic upstream acceptor site (ΔAG = .61 at −23 bp), consistent with exon skipping or aberrant splicing (Figure 2D, Supporting Information). No additional pathogenic variants were identified in epilepsy‐ and/or neurodevelopmental disorder‐related genes.

To evaluate whether the NM_020708.5:c.53‐2A>C variant affects *SLC12A5* splicing, we performed a minigene splicing assay, a widely used approach when patient‐derived RNA is unavailable. The assay demonstrated a dual splicing defect: a major out‐of‐frame exon 2 skipping event and a minor activation of a cryptic intraexonic acceptor site resulting in a 17‐bp deletion (Figures S3–S5).

Both aberrant transcripts introduce frameshifts and premature termination codons and are therefore predicted to undergo nonsense‐mediated mRNA decay, leading to functional haploinsufficiency in this highly loss of function‐intolerant gene (probability of being loss of function intolerant = 1).

The second variant is a single‐nucleotide substitution in exon 18, corresponding to NM_001134771.2:c.2366T>G (hg19) and resulting in NM_001134771.2:c.2366T>G (p.Leu789Arg) in KCC2a and NM_020708.5:c.2297T>G (p.Leu766Arg) in KCC2b. Each variant was inherited from an asymptomatic parent (Figure 2A,B).

In both KCC2a and KCC2b proteins, the affected leucine residue is located within the intracellular *C*‐terminal domain. In silico predictions were in favor of a deleterious effect (CADD, Combined Annotation Dependant Depletion) score = 26.5, AlphaMissense = .965, REVEL, Rare Exome Variant Ensemble Leraner score = .926. Structural modeling suggested an altered surface potential of KCC2 from a hydrophobic patch into a positively charged surface (Figure 2C, c.5 and c.6).

### 3.3 In vitro analyses

#### 3.3.1 Exogenously expressed KCC2 variants

KCC2 is a K^+^/Cl^−^ cotransporter critically involved in the maintenance of neuronal Cl^−^ homeostasis that regulates the inhibitory strength of GABA and glycine neurotransmission.^1^ The reduction of KCC2 ion transport activity is associated with enhancement of neuronal network spiking and different types of epilepsy.^16^ To directly assess whether single amino acid substitutions in identified variants alter KCC2 function, we generated cDNA constructs encoding the nonmutated (WT) human KCC2b protein (hereafter referred to as KCC2_WT_) as well as KCC2_WT_‐derived constructs harboring the Phe117Ile (KCC2_F117I_), Arg420Cys (KCC2_R420C_), and Leu766Arg (KCC2_L766R_) substitutions. For heterologous expression experiments, we used the KCC2b splice variant, the predominant form in mature neurons, which exhibits intrinsic ion transport properties comparable to those of KCC2a when expressed in heterologous systems.^17^, ^18^

**FIGURE 3:**
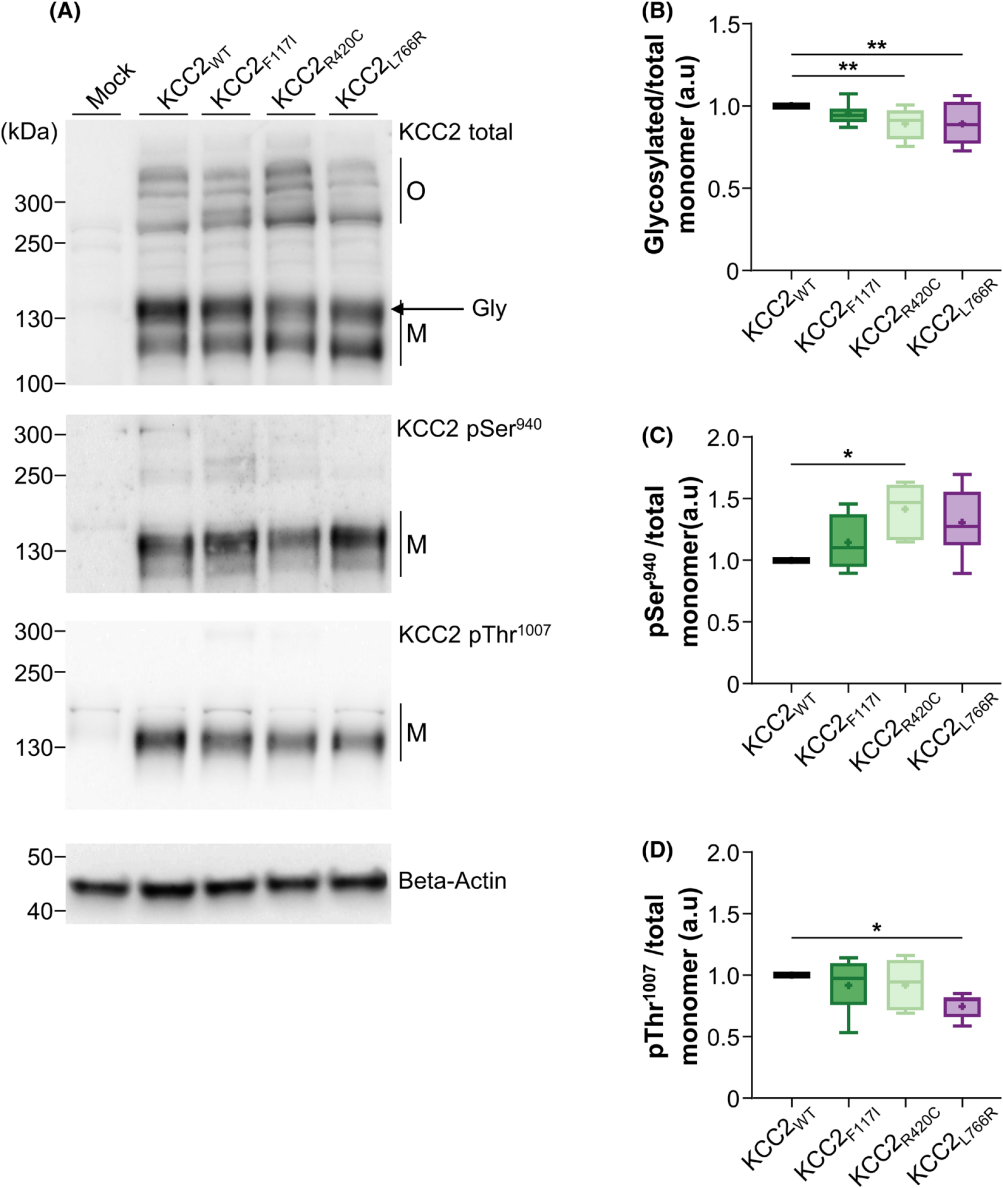
Biochemical properties of pathogenic variants overexpressed in HEK293 cells. (A) Western blot analysis of HEK293 cells expressing either empty vector or the indicated KCC2 variants. Each panel shows a separate Western blot membrane, loaded with an equal amount of cell lysates and probed with the indicated antibodies (see Supporting Information Methods). Letters O and M indicate oligomeric and monomeric forms, respectively. “Gly” and arrow indicate the band corresponding to the glycosylated form of KCC2. The bottom panel shows beta‐actin immunoreactivity revealed on the KCC2 total Western blot membrane. Similar images of beta‐actin were obtained on membranes probed with anti‐KCC2 pSer940 and pThr1007 antibodies. (B) Boxplots summarizing quantitative immunoblot analysis of the ratio of glycosylated to total KCC2 monomers. Values were normalized to the mean of KCC2_WT_ for clarity. A Friedman analysis of variance test revealed a significant difference in glycosylation among the four variants, *χ* ^2^ (3) = 15.00, *p* = .002. Post hoc Dunn test showed no significant change in glycosylation for KCC2_F117I_ (*p* = .644), but a significant reduction for KCC2_R420C_ and KCC2_L766R_ (*p* = .002 and *p* = .003, respectively, *N* = 7; *n =* 14). In this and subsequent figures, “*N*” refers to the number of independent cell culture preparations; “*n*” indicates the number of values (technical duplicates). Asterisks denote statistical significance: *p < .05, **p < .01. In boxplots, boxes span the interquartile range (Q1–Q3), the line inside indicates the median, “+” shows the mean, and whiskers represent the minimum and maximum. (C, D) Boxplots of quantitative immunoblot analysis showing the ratio of phosphorylated pSer940 (C) and pThr1007 (D) to total KCC2 monomers, normalized to the mean of KCC2_WT_. *N* = 4; *n* = 6 for pSer940 and *N* = 4; *n* = 7 for pThr1007. See Table S4 for exact values and statistical comparisons WT, wild type.

Western blotting of generated KCC2b variants overexpressed in HEK293 cells revealed a typical multiband migration pattern^19^ for all variants, including a ~120‐ and 140‐kDa double band of the monomer and >250‐kDa bands of oligomers (Figure 3A). The upper monomer band reflects glycosylated KCC2, a posttranslational modification essential for transporter function. Quantitative analysis of the glycosylated KCC2 form revealed a statistically significant decrease in glycosylation for KCC2_R420C_ and KCC2_L766R_, but not KCC2_F117I_ (Figure 3A,B). For this and all subsequent statistical plots, see figure legends and Supporting Information Tables S4–S8 for the number of experiments, as well as *Q*‐ and *p*‐values.

Phosphorylation is a key posttranslational regulator of KCC2 membrane trafficking and chloride transport activity.^16^, ^20^ Previous mass spectrometry and mutation analyses in HEK293 cells have identified multiple putative phosphorylation sites on the KCC2b isoform^21^, ^22^; however, quantitative assessment of site‐specific phosphorylation remains technically limited by the availability and validation of high‐affinity phosphospecific reagents. We therefore focused our investigation on Ser940 and Thr1007, two functionally well‐characterized residues for which validated phosphospecific antibodies are available.^20^

Relative to KCC2_WT_, the phosphorylation of Ser940 was not significantly different in KCC2_F117I_ and KCC2_L766R_ variants but showed a significant increase in KCC2_R420C_ variant (Figure 3A,C). Thr1007 phosphorylation was significantly reduced in KCC2_L766R_ variant compared to KCC2_WT_ but remained unaltered in KCC2_F117I_ and KCC2_R420C_ (Figure 3A,D).

#### 3.3.2 Ion transport activity and surface expression

##### Case A variants

**FIGURE 4:**
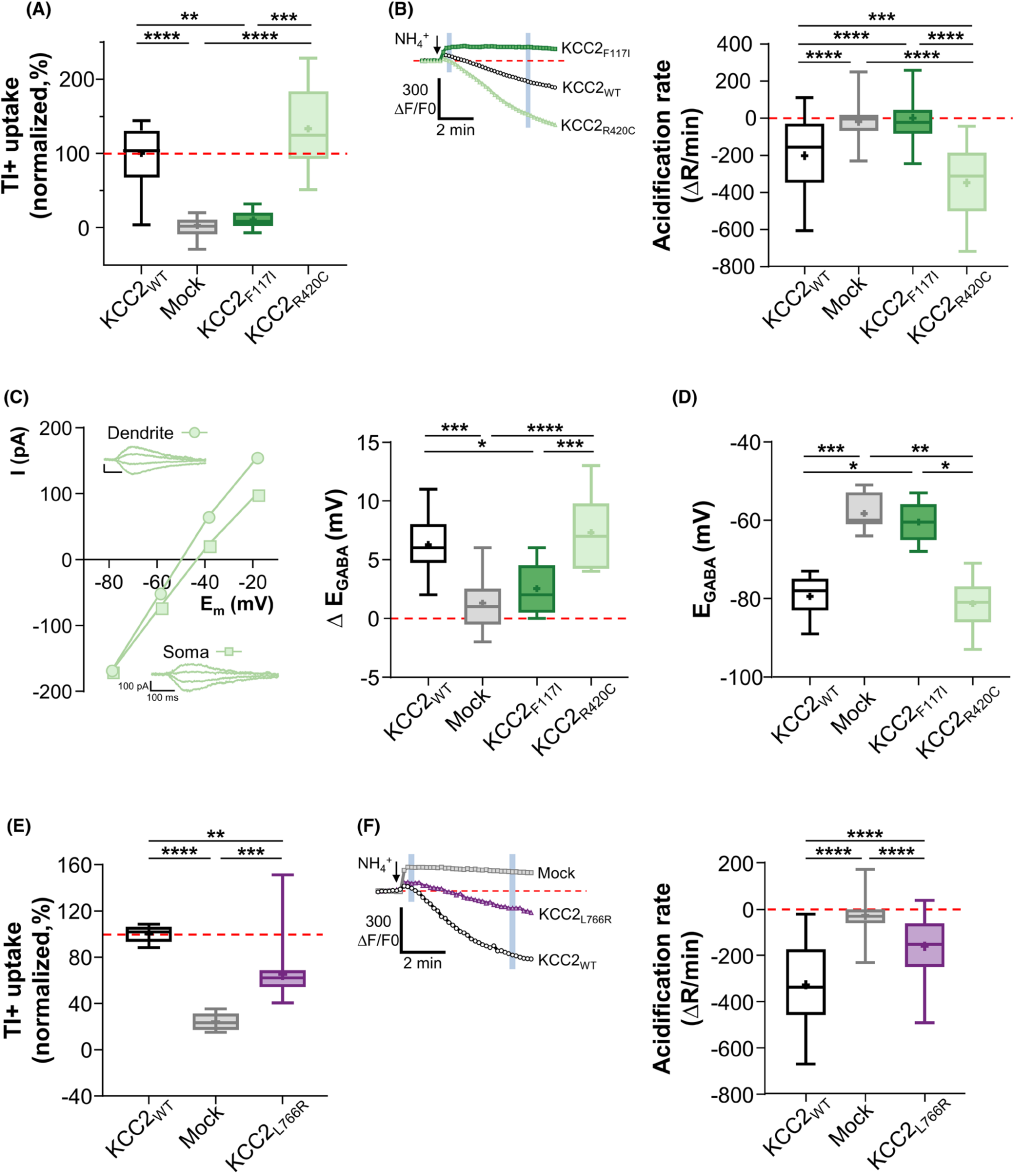
Ion transport activity of overexpressed KCC2_F117I_, KCC2_R420C_, and KCC2_L766R_ variants. (A) Tl^+^ uptake in HEK293 cells transiently transfected with vectors encoding KCC2_WT_, KCC2_F117I_, KCC2_R420C_, or empty vector (Mock). *N* = 7 independent cell culture preparations; *n* = 27 for KCC2_WT_ and Mock, *n* = 26 for KCC2_R420C_, *n* = 21 for KCC2_F117I_. “*n*” represents the number of multiplate wells. Kruskal–Wallis test was followed by Dunn post hoc test. (B) NH_4_^+^‐induced and KCC2‐dependent changes of pH_i_ in N2a cells transiently cotransfected with pH sensor and indicated KCC2 variants. Inset on the left illustrates timeline plots of the NH_4_^+^‐induced pH_i_ changes in cells expressing KCC2_WT_, KCC2_F117I_, and KCC2_R420C_. Kruskal–Wallis test was followed by Dunn post hoc test; *N* = 6; *n* = 107 for KCC2_WT_, *n* = 75 for Mock, *n* = 42 for KCC2_F117I_, *n* = 53 for KCC2_R420C_; “*n*” represents the number of cells. (C) Cl^−^ extrusion capacity of 6–8 days in vitro (DIV) cultured rat hippocampal neurons coexpressing enhanced green fluorescent protein and indicated KCC2b constructs was quantified as the somatodendritic E_GABA_ gradient (ΔE_GABA_ = E_GABA‐soma_ – E_GABA‐dendrite_). Inset on the left illustrates whole‐cell patch‐clamp recordings and corresponding current–voltage curve of GABA_A_ receptor‐mediated currents (I_GABA_) induced by focal applications of isoguvacine to the soma and dendrite of KCC2_R420C_ neuron. Kruskal–Wallis test was followed by Dunn post hoc test; *n* = 18 for KCC2_WT_, *n* = 13 for Mock, *n* = 13 for KCC2_F117I_, *n* = 20 for KCC2_R420C_; “*n*” represents the number of recorded cells from *N* = 4 independent cultures. (D) E_GABA_ in 6–8 DIV cultured rat hippocampal neurons measured using gramicidin‐perforated patch‐clamp recording. Kruskal–Wallis test was followed by Dunn post hoc test; *N* = 4; *n* = 7 for KCC2_WT_, *n* = 7 for Mock, *n* = 6 for KCC2_F117I_, *n* = 7 for KCC2_R420C_. (E) Tl^+^ uptake in HEK293 cells transfected with KCC2_WT_, empty vectors (Mock), or KCC2_L766R_ variant from Case B. *N* = 4; *n* = 12 for KCC2_WT_ and Mock, *n* = 24 for KCC2_L766R_. *n* represents the number of multiplate wells. Kruskal–Wallis test was followed by Dunn post hoc test. (F) NH_4_^+^‐induced and KCC2‐dependent changes of pH_i_ in N2a cells cotransfected with pH sensor and Case B KCC2_L766R_ variant as well as respective control vectors, KCC2_WT_, and Mock. *N* = 3; *n* = 60 for KCC2_WT_, *n* = 47 for Mock, *n* = 76 for KCC2_L766R_. Comparison was made using Kruskal–Wallis test followed by Dunn post hoc test. See Table S5 for exact values and statistical comparisons. *p < .05, **p < .01, ***p < .001, ****p < .0001. GABA, γ‐aminobutyric acid; WT, wild type.

Two distinct in vitro assays measuring the ion transport activity of KCC2 (Tl^+^ and NH4^+^ tests) revealed complete silencing of KCC2_F117I_ overexpressed in HEK293 or N2a cells (Figure 4A,B). Contrary to KCC2_F117I_, the KCC2_R420C_ variant, when compared to KCC2_WT_, showed a trend toward increased ion transport in HEK293 cells (Figure 4A) and a significant gain of function in N2a cells (Figure 4B). The absence of reduced ion transport in the KCC2_R420C_ variant was unexpected given the severe pathology in the patient. We, therefore, next studied whether the KCC2_R420C_ function is preserved in a neuronal environment. KCC2_R420C_ overexpressed in immature cultured rat hippocampal neurons exhibited Cl^−^ extrusion ability comparable to WT KCC2 in the soma–dendrite gradient test^23^ (Figure 4C) and, similar to KCC2_WT_, generated negative reversal potentials of GABA_A_ receptor‐mediated current, reflecting reduced intraneuronal Cl^−^ levels, as measured using gramicidin‐perforated patch‐clamp recordings^24^ (Figure 4D). Contrary to KCC2_R420C_ and consistent with results obtained in HEK293 and N2a cells, the variant KCC2_F117I_ was inactive when overexpressed in immature neurons.

To assess whether Phe117Ile and Arg420Cys variants affected surface expression, we cotransfected N2a cells with KCC2 constructs and pEYFP‐Mem, a membrane‐targeted marker (Figure S6A). KCC2_WT_ and both variants showed a fluorescence signal located both at the membrane and in the cytoplasm. As a negative control, we used the KCC2_CTD_ construct, which encodes only the intracellular *C*‐terminal domain, lacks transmembrane domains, and thus remains cytoplasmic. Quantification of the membrane‐to‐cytoplasm fluorescence ratio revealed no significant difference between KCC2_WT_ and the variants. However, both KCC2_F117I_ and KCC2_R420C_ constructs showed significantly higher membrane localization than KCC2_CTD_ (Figure S6B,C). Thus, the Phe117Ile mutation renders KCC2 inactive without affecting its surface expression, whereas the Arg420Cys substitution does not impair and may even increase KCC2's ion transport ability.

##### Case B KCC2_L766R_ variant

Both the Tl^+^ flux test in HEK293 cells and the ammonium test in N2a cells revealed partial inhibition of the ion transport ability of the KCC2_L766R_ variant; its activity was significantly decreased as compared with WT KCC2 but was higher than in mock‐transfected cells (Figure 4E,F).

The analysis of the KCC2_L766R_ variant's surface expression revealed no changes as compared to KCC2_WT_ but showed a significant difference relative to the KCC2_CTD_ construct (Figure S6C).

#### 3.3.3 Excitatory synapse formation

Previous studies showed that overexpression of KCC2b constructs carrying the Cys568Ala or Arg592His mutations in immature neurons markedly decreases miniature excitatory postsynaptic current (mEPSC) frequency and dendritic spine density compared with WT KCC2.^4^, ^25^ Based on these observations, we overexpressed KCC2_WT_, KCC2_F117I_, or KCC2_R420C_ in cultured rat hippocampal neurons at an early developmental stage, characterized by low endogenous KCC2 expression, at 6 days in vitro (DIV).^26^ Neurons were then allowed to form synapses in the presence of ectopic KCC2 variants, and mEPSCs and dendritic spines were analyzed 8 days later (14 DIV).

**FIGURE 5:**
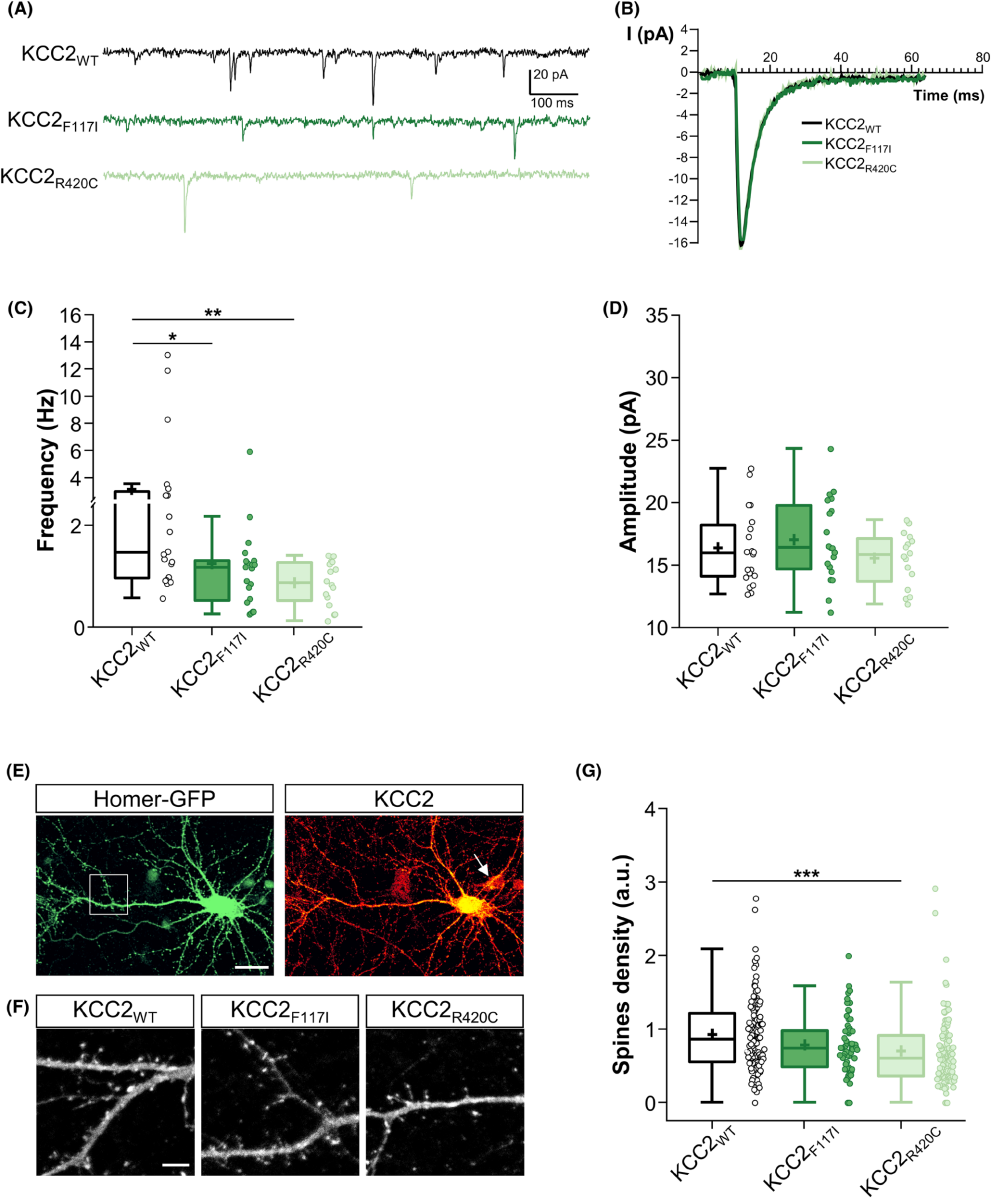
Excitatory synapse formation in pyramidal neurons of primary rat hippocampal cultures transfected with vectors expressing KCC2_WT_, KCC2_F117I_, or KCC2_R420C_ variants. (A) Representative traces of miniature excitatory postsynaptic currents (mEPSCs) recorded from pyramidal neurons expressing KCC2_WT_, KCC2_F117I_, or KCC2_R420C_. (B) Superimposed average traces of individual mEPSCs recorded from neurons expressing the indicated KCC2 variant. Each trace represents the mean signal from 10–16 neurons (eight different cultures), with 100 mEPSCs analyzed per neuron. (C, D) Boxplots showing mEPSC frequencies (C) and amplitudes (D). *N* = 8 independent cell culture preparations; *n* = 19 for KCC2_WT_, *n* = 20 for KCC2_F117I_, *n* = 16 for KCC2_R420C_. Kruskal–Wallis test was followed by Dunn post hoc test. (E) Representative image of a neuron cotransfected with Homer1b–green fluorescent protein (GFP) and KCC2_F117I_, visualized using anti‐GFP and anti‐KCC2 antibodies. The white square in the Homer1b‐GFP image marks the dendritic region where spine density was quantified. The arrow in the KCC2 image indicates a nontransfected neuron, highlighting differences in exogenous versus endogenous KCC2 expression. Scale bar: 20 μm. (F) Representative images of dendritic spines in 14 days in vitro cultured rat hippocampal neurons expressing the indicated KCC2 variants. Scale bar: 4 μm. (G) Boxplots of spine densities on secondary dendrites of pyramidallike neurons. Data were collected from 5 cultures (*N*), analyzing 10–12 neurons per culture and 2–3 dendritic segments (40 μm each) per neuron. *n* denotes the number of analyzed segments. *n* = 116 for KCC2_WT_, *n* = 62 for KCC2_F117I_, *n* = 120 for KCC2_R420C_. Kruskal–Wallis test was followed by Dunn post hoc test. See Table S6 for exact values and statistical comparisons. *p < .05, **p < .01, ***p < .001. WT, wild type.

Pyramidal KCC2_WT_‐expressing neurons displayed a wide range of mEPSC frequencies (.9–13 Hz), whereas KCC2_R420C_‐expressing neurons showed consistently low values significantly different from KCC2_WT_ values (<1.5 Hz). KCC2_F117I_‐expressing neurons exhibited intermediate values that were also significantly lower as compared to KCC2_WT_ (Figure 5). No significant changes were observed in mEPSC decay kinetics (Figure 5B) or amplitudes (Figure 5D), suggesting a reduction in synapse number rather than changes in postsynaptic receptor composition.

To visualize dendritic spines, neurons were cotransfected with Homer1b‐GFP, which labels postsynaptic densities without affecting spine morphology.^27^ Confocal analysis revealed a significant reduction in spine density in KCC2_R420C_‐expressing neurons compared to KCC2_WT_ (Figure 5E–G). Spine density in KCC2_F117I_ ‐expressing cells did not differ from controls.

#### 3.3.4 Mixture of KCC2_F117I_ and KCC2_R420C_ cDNAs

**FIGURE 6:**
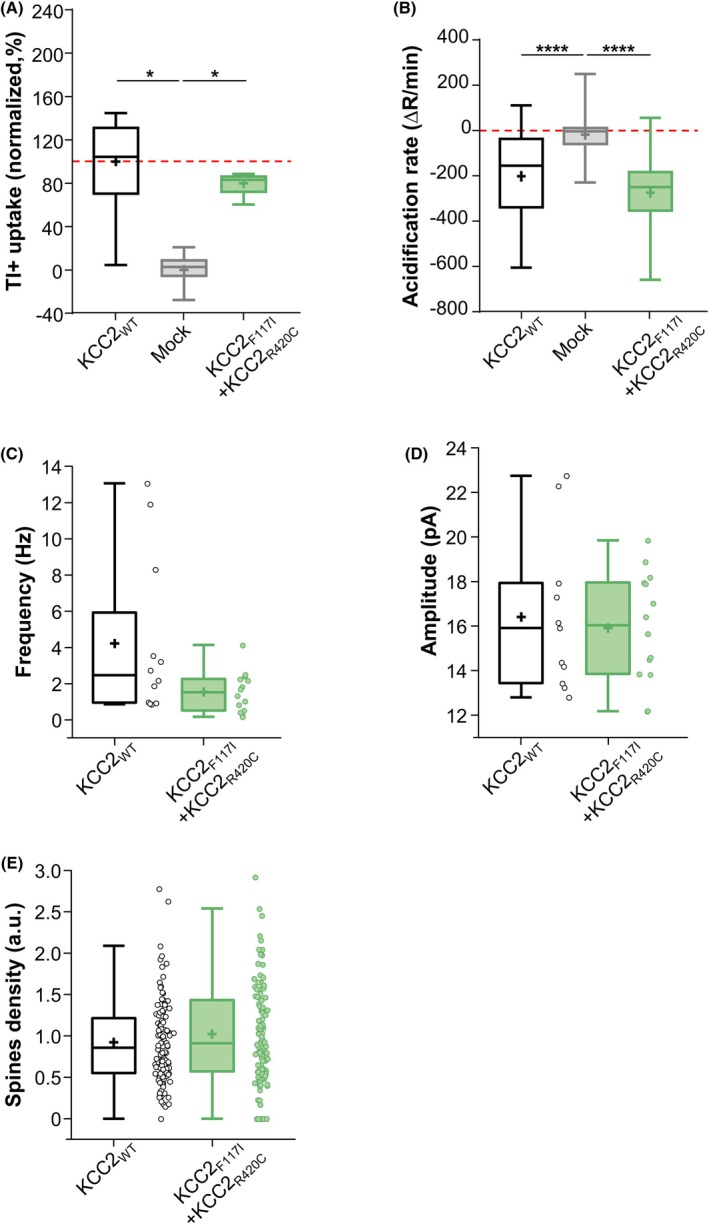
In vitro analysis of the properties of coexpressed KCC2_F117I_ plus KCC2_R420C_ variants in 1:1 ratio. (A) Boxplots show Tl^+^ uptake in HEK293 cells transiently transfected with KCC2_WT_, KCC2_F117I_+KCC2_R420C_, and Mock. *N* = 5 independent cell culture preparations. *n* = 27 for KCC2_WT_ and Mock, *n* = 15 for KCC2_F117I_ + KCC2_R420C_. (B) NH_4_^+^‐mediated acidification rates in N2a cells. *N* = 6; *n* = 107 for KCC2_WT_, *n* = 75 for Mock, *n* = 36 for KCC2_F117I_ +KCC2_R420C_. The data in panels A and B were compared using Kruskal–Wallis test followed by Dunn post hoc test. (C, D) Frequency (C) and amplitude (D) of miniature excitatory postsynaptic currents recorded from neurons expressing KCC2_WT_ or KCC2_F117I_+KCC2_R420C_. *N* = 8; *n* = 19 for KCC2_WT_, *n* = 14 for KCC2_F117I_+KCC2_R420C_. Mann–Whitney *U*‐test. (E) Spines density on secondary dendrites of pyramidallike neurons expressing KCC2_WT_ or KCC2_F117I_+KCC2_R420C_. *N* = 5; *n* = 116 for KCC2_WT_, *n* = 109 for KCC2_F117I_+KCC2_R420C_. Mann–Whitney *U*‐test. See Table S7 for exact values and statistical comparisons. *p < .05, ****p < .0001. WT, wild type.

To mimic the composite heterozygous composition of KCC2_F117I_ and KCC2_R420C_ variants found in the patient, we transfected the cell lines with a .5:.5 mixture of cDNAs encoding KCC2_F117I_ and KCC2_R420C_ and compared the properties of this composite transporter with KCC2_WT_. The tests of KCC2 functionality (Tl^+^ and NH_4_^+^ tests) revealed no statistically significant difference between KCC2_WT_ and KCC2_F117I_ plus KCC2_R420C_ mixture (Figure 6A,B). Similarly, no statistically significant difference was observed in the frequency and amplitude of mEPSCs (Figure 6C) or density of dendritic spines (Figure 6D).

## 4 DISCUSSION

This study reports two novel cases of severe DEE caused by biallelic variants in *SLC12A5*, encoding the K^+^/Cl^−^ cotransporter KCC2. The clinical presentation showed early onset, refractory epilepsy with progressive neurological deterioration and early mortality. Functional characterization of the identified variants clarifies their pathogenic mechanisms and highlights the essential role of KCC2 in neuronal development and function.

### 4.1 Clinical implications

Both cases exhibited profound neurological impairment from birth, with refractory seizures despite extensive trials of multiple ASMs, vagus nerve stimulation, and dietary therapy. The striking clinical overlap between the two cases, including severe hypotonia, respiratory fragility, and global developmental delay, highlights the devastating nature of *SLC12A5*‐related DEE.

The clinical phenotypes closely resemble those of previously reported patients with *SLC12A5* pathogenic variants, including two individuals with homozygous variants^7^, ^10^ and seven carrying compound heterozygous missense variants^7^, ^8^, ^9^ (Table S1). All reported children presented with EIMFS, accompanied by developmental regression or severe global developmental delay. Only a few demonstrated neurodevelopmental progress, primarily during periods of improved seizure control. The neurological comorbidities included microcephaly and hypotonia.

A particularly concerning aspect of this condition is its high lethality. Among the 11 reported patients with biallelic *SLC12A5* variants, four (36%) have died (two previously reported and two from our study) at the time of publication, underscoring the severe prognosis associated with *SLC12A5*‐related DEE.

The progressive brain atrophy observed in Case A by 2 years of age, along with similar findings in previous reports,^7^, ^10^ indicates that *SLC12A5* dysfunction may contribute to neurodegenerative processes beyond epileptogenesis. This aligns with previous studies suggesting that KCC2 plays a role in neuronal survival and synaptic plasticity,^28^ further implicating its dysfunction in widespread neurodevelopmental and neurodegenerative pathology. Additionally, Case A demonstrated a progressive loss of previously acquired motor and interactional abilities, ultimately becoming unable to sit, grasp objects, or maintain eye contact. This trajectory exceeds the static “severe developmental delay” phenotype most commonly reported in the literature.^7^, ^8^, ^9^, ^10^

Recurrent bone fractures without documented trauma, observed in Case A, represent a previously unreported clinical finding in *SLC12A5*‐related DEE. Although we do not claim a direct causal relationship, we discuss potential contributing factors, including chronic hypotonia, severe immobility, and the possibility of underrecognized, noncanonical roles of KCC2. This feature is presented cautiously as an observation warranting future investigation rather than a defining phenotypic hallmark.

### 4.2 Genetic and molecular mechanisms

Structural and in vitro analyses of the four variants demonstrate that they impair KCC2 function through distinct mechanisms, expanding the spectrum of pathogenic effects beyond those previously described in biallelic missense cases.^7^, ^8^, ^9^ These include a variant with no altered ion transport function, and a nonsense‐mediated decay (NMD)‐inducing splice‐site variant leading to allelic silencing.

#### 4.2.1 Case A

Both missense variants affect residues located in functionally relevant transmembrane regions, and in silico modeling predicts structural disruption. The absence of overt neurological disease in heterozygous carrier parents supports the possibility that pathogenicity requires compound heterozygosity and reflects the combined effects of the two variants.

In vitro assays showed that the Phe117Ile variant produces a near‐complete loss of ion transport. This result is consistent with structural predictions placing this residue near the K^+^ binding site and aligns with prior reports linking KCC2 loss of function to epilepsy phenotypes.^28^

In contrast, the Arg420Cys variant, compared to KCC2_WT_, did not exhibit a detectable decrease in ion transport in HEK293 cells or primary cultured neurons and even exhibited a gain of function when expressed in N2A cells. A pathogenic mechanism driven primarily by gain of function appears less likely, as previous studies reported that homozygous knockin mice carrying a constitutively active KCC2 variant (Thr906Ala/Thr1007Ala) do not show overt deregulation of neuronal network activity and instead exhibit reduced susceptibility to chemoconvulsant‐induced seizures.^29^

An alternative suggestion is that pathogenicity occurs on the level of noncanonical action involving interaction of the large *C*‐terminus of KCC2 with intracellular partners.^3^ This hypothesis is supported by evidence of altered Ser940 phosphorylation and by synaptic phenotypes including changes in dendritic spine development and mEPSC frequency.

KCC2 participates in a large neuronal interactome and interacts with multiple components of excitatory postsynaptic density.^30^ Neuronal cultures derived from KCC2 knockout mice, which die perinatally, show marked defects in synapse and spine maturation, suggesting a developmental role for KCC2 beyond chloride extrusion.^31^

Several overexpression studies have reported that KCC2 promotes spine and synapse formation both in KCC2‐deficient cultures^31^ and in WT fish^32^ or rat embryos^4^, ^25^ at early developmental stages when endogenous KCC2 expression is low. In contrast to KCC2_WT_, at least two KCC2 variants with abrogated ion transport activity (Cys568Ala and Arg592His) did not promote glutamatergic synaptogenesis when overexpressed in immature neurons.^4^, ^25^, ^32^ Reynolds et al.^32^ attributed this lack of effect to impaired ion transport and altered GABAergic signaling, whereas Kaila and colleagues favored an ion transport‐independent hypothesis.^4^, ^25^ The Arg420Cys variant identified in the present study, which, unlike Cys568Ala and Arg592His, preserves ion transport function in vitro, may represent a useful tool to explore the ion transport‐independent functions of KCC2 and to directly test these noncanonical mechanisms.

Moreover, it provides an opportunity to generate a knockin animal model to support or refute the noncanonical ion transport hypothesis under endogenous expression conditions. This is particularly important, because much of the existing evidence relies on overexpression paradigms, in which KCC2_WT_ overexpression has been reported to be developmentally disruptive in vivo, including malformations and early developmental perturbances in fish^32^ or rat^33^ embryos.

Finally, the mixed functional profile observed in Case A highlights the need for endogenous expression systems. Our coexpression experiments did not show clear additive or intermediate phenotypes, likely reflecting limitations of heterologous systems (see Supporting Information). Future studies using patient‐derived induced pluripotent stem cell neurons and knockin models will be essential to determine whether Arg420Cys affects KCC2 regulatory signaling, protein interactions, or synaptic development in vivo, and to define how these effects interact with the severe transport deficit caused by Phe117Ile.

#### 4.2.2 Case B

The splice acceptor variant c.53‐2A>C was shown by the minigene assay to disrupt normal splicing, resulting in out‐of‐frame exon skipping and cryptic splice‐site usage. Both aberrant transcripts introduce premature termination codons located within an NMD‐sensitive region, strongly predicting degradation of the mutant mRNA and functional loss of expression from the affected allele.

The second variant, Leu766Arg, affected the intracellular *C*‐terminal regulatory domain and resulted in reduced glycosylation, decreased Thr1007 phosphorylation, and partial loss of ion transport function. The reduced glycosylation is consistent with impaired surface delivery of KCC2,^19^ whereas the altered phosphorylation may reflect either compensatory regulation or altered interaction with kinases or phosphatases.

Taken together, Case B illustrates a particularly severe biallelic pathogenic mechanism, combining complete functional inactivation of one allele due to NMD with partial loss of function of the second allele. This genetic configuration provides a compelling explanation for the severe epileptic encephalopathy observed and contrasts with the unaffected heterozygous parent, in whom expression from the intact allele is preserved and likely sufficient to maintain normal KCC2 functional activity.

### 4.3 Pathophysiological insights

The critical role of KCC2 in maintaining neuronal chloride homeostasis is well established, and its dysfunction has been linked to hyperexcitability and epileptogenesis. Our findings reinforce the concept that both loss of KCC2 ion transport activity and disruption of its regulatory functions can result in profound neurological disease.

Importantly, the divergent functional effects observed across variants highlight that disease severity cannot be predicted solely by ion transport measurements, underscoring the multifaceted roles of KCC2 in neuronal development and network stability.

### 4.4 Therapeutic considerations

The extreme resistance to ASMs observed in both cases underscores the urgent need for novel therapeutic strategies targeting KCC2 dysfunction. Approaches such as pharmacological modulation of KCC2 activity, gene‐based therapies, or variant‐specific precision medicine strategies merit further exploration. The partial responsiveness to the ketogenic diet observed in Case B and previous reports^8^, ^9^ suggests that metabolic interventions may offer limited symptomatic benefit in select patients.

## 5 CONCLUSIONS

Our study expands the mutational and mechanistic spectrum of *SLC12A5*‐related DEE and demonstrates that both loss‐ and dysregulation‐of‐function mechanisms can converge on a severe epileptic encephalopathy phenotype. The findings underscore the indispensable role of KCC2 in early brain development and highlight the need for disease models that accurately reflect endogenous gene expression to guide future therapeutic strategies.

## AUTHOR CONTRIBUTIONS

*Conception and design of the study:* Mira Hamze, Christophe Porcher, Gaetan Lesca, and Igor Medina. *Acquisition and/or analysis of data:* Mira Hamze, Robyn Whitney, Dorothée Ville, Nathalie Villeneuve, Anna‐Maria Hartmann, Lisa Becker, Jens Hausmann, Jinwei Zhang, Cathy Brier, Lucie I. Pisella, Perrine Friedel, Audrey Labalme, Eudeline Alix, Nicolas Chatron, Damien Sanlaville, Sylvie Gory‐Fauré, Eric Denarier, Christophe Porcher, Gaetan Lesca, and Igor Medina. *Drafting substantial portions of the manuscript or figures:* Mira Hamze, Robyn Whitney, Anna‐Maria Hartmann, Jens Hausmann, Christophe Porcher, Gaetan Lesca, and Igor Medina. All authors approved the final version and agree to be held accountable for all aspects of the work.

## FUNDING INFORMATION

This research was funded by the National Institute of Health and Medical Research, the National Center for Scientific Research, the French National Agency for Research (grant number R07066AS 2008‐2011 to M.H., C.B., C.P., G.L., I.M.), the French Foundation of Epilepsy Research (to L.I.P., G.L., I.M.), and the French Ministry of Education (to L.I.P., M.H.). It received support from the French government under the France 2030 investment plan, as part of the Initiative d'Excellence d'Aix‐Marseille Université—A*MIDEX (AMX‐19‐IET‐007) through the Marseille Maladies Rares Institute. This work was also supported by the INMED core facility PBMC.

## CONFLICT OF INTEREST STATEMENT

None of the authors has any conflict of interest to disclose. We confirm that we have read the Journal's position on issues involved in ethical publication and affirm that this report is consistent with those guidelines.

## ETHICS STATEMENT

The case was managed and reported in compliance with institutional ethical standards and the ethical principles outlined in the French and Canadian Bioethics laws. Formal ethics committee approval was not required for publication of this individual case report.

## PATIENT CONSENT STATEMENT

Written informed consent was obtained from the caregivers of the patients in accordance with local university/hospital ethics procedures and the French and Canadian Bioethics laws.

## Supporting information


**Figure S1.** Case A. Different types of multifocal seizures recorded at ages 7 months (A, B) and 16 months (C) are shown.
**Figure S2.** Case B. Consecutive electroencephalograms recorded during the first week of life are shown.
**Figure S3.** Minigene splicing assay for the *SLC12A5* c.53‐2A>C.
**Figure S4.** Real‐time polymerase chain reaction and TOPO TA product Sanger sequencing.
**Figure S5.** Schematic representation of real‐time polymerase chain reaction products from minigene experiments in HeLa cells shown in Figure S3.
**Figure S6.** Surface expression of different variants of KCC2 cotransfected with a fragment of neuromodulin linked to enhanced yellow–green fluorescent protein (pEYFP‐Mem).
**Table S1.** Clinical characteristics of patients with pathogenic *SLC12A5* variants.
**Table S2.** In silico splice prediction analysis of the *SLC12A5* variant NM_020708.5:c.53‐2A>C affecting a shared canonical splice acceptor site.
**Table S3.** In silico splice prediction analysis of the *SLC12A5* variant. NM_001134771.2:c.122‐2A>C.
**Table S4.** Summary of statistical analyses for Western blot data presented in Figure 3.
**Table S5.** Summary of statistical analyses for ion transport ability data presented in Figure 4.
**Table S6.** Summary of statistical analyses of miniature excitatory postsynaptic current parameters presented in Figure 5.
**Table S7.** Summary of statistical analyses for results from cells coexpressing the Phe117Ile and Arg420Cys variants in Figure 6.
**Table S8.** Summary of statistical analyses of cell surface expression data presented in Figure S6.

## Data Availability

Data are available upon reasonable request to the corresponding author.
