# Supplementary material for: Compound heterozygous SLC12A5 variants expand the molecular and functional spectrum of KCC2‐developmental and epileptic encephalopathy
Source: Epilepsia. 2026 Apr 25;67(7):3657–73. doi: 10.1002/epi.70258 (PMC13361015; doi:10.1002/epi.70258)
Supplement: Supplementary file 1 — Figure S1. Case A. Different types of multifocal seizures recorded at ages 7 months (A, B) and 16 months (C) are shown. Figure S2. Case B. Consecutive electroencephalograms recorded during the first week of life are shown. Figure S3. Minigene splicing assay for the SLC12A5 c.53‐2A>C. Figure S4. Real‐time polymerase chain reaction and TOPO TA product Sanger sequencing. Figure S5. Schematic representation of real‐time polymerase chain reaction products from minigene experiments in HeLa cells shown in Figure S3. Figure S6. Surface expression of different variants of KCC2 cotransfected with a fragment of neuromodulin linked to enhanced yellow–green fluorescent protein (pEYFP‐Mem). Table S1. Clinical characteristics of patients with pathogenic SLC12A5 variants. Table S2. In silico splice prediction analysis of the SLC12A5 variant NM_020708.5:c.53‐2A>C affecting a shared canonical splice acceptor site. Table S3. In silico splice prediction analysis of the SLC12A5 variant. NM_001134771.2:c.122‐2A>C. Table S4. Summary of statistical analyses for Western blot data presented in Figure 3. Table S5. Summary of statistical analyses for ion transport ability data presented in Figure 4. Table S6. Summary of statistical analyses of miniature excitatory postsynaptic current parameters presented in Figure 5. Table S7. Summary of statistical analyses for results from cells coexpressing the Phe117Ile and Arg420Cys variants in Figure 6. Table S8. Summary of statistical analyses of cell surface expression data presented in Figure S6. [file EPI-67-3657-s001.pdf]

Supplementary Material:

Title: Compound Heterozygous *SLC12A5* Variants Expand the Molecular and Functional Spectrum of KCC2-Related Developmental and Epileptic Encephalopathy

Running head: Severe KCC2 DEE

Mira Hamze, PhD, Robyn Whitney, MD, Dorothée Ville, MD, Nathalie Villeneuve, MD, Anna-Maria Hartmann, PhD, Lisa Becker, PhD, Jens Hausmann, PhD, Jinwei Zhang, PhD, Cathy Brier, MS, Lucie I. Pisella, PhD, Perrine Friedel, PhD, Audrey Labalme, PhD, Eudeline Alix, PhD, Nicolas Chatron, PhD, Damien Sanlaville, PhD, Sylvie Gory-Fauré, PhD, Eric Denarier, PhD, Christopher Porcher, PhD, Gaetan Lesca, MD, PhD and Igor Medina, PhD

Contents

Supplementary Methods: ... 4
EEG recordings ... 4
Genetic testing and data analysis ... 4
Minigene splicing reporter assay ... 5
Expression constructs ... 5
Chemicals. ... 6
Neuronal and Cell line cultures. Transfection. ... 6
Patch-clamp recordings. ... 7
Soma-dendrite gradient Cl^- extrusion assay ... 7
Gramicidin perforated patch Clamp recording ... 8
mEPSCs recordings ... 8
Western blot analysis ... 8
Antibodies for immunocytochemistry ... 9
Tl^+ flux assay ... 9
$NH4^+$ flux assay ... 10
Surface expression analysis ... 10
Statistical analysis ... 11
Supplementary Results ... 11
Antiseizure treatment ... 11
Case A. ... 11
Case B. ... 12
In-silico analysis: ... 12
Case A. ... 12
Case B. ... 12
Supplementary Figures: ... 14
Supplementary Figure S1: ... 14
Supplementary Figure S2: ... 16
Supplementary Figure S3: ... 17
Supplementary Figure S4: ... 18
Supplementary Figure S5: ... 19
Supplementary Figure S6: ... 20
Supplementary Tables: ... 21
Supplementary Table S1. ... 21

Supplementary Table S2. 23
Supplementary Table S3. 23
Supplementary Table S4. 24
Supplementary Table S5. 25
Supplementary Table S6. 26
Supplementary Table S7. 26
Supplementary Table S8. 27
References 28

Supplementary Methods:

EEG recordings

Case A. The patient's EEG was recorded using a conventional commercial Micromed® setup (Micromed, France) with the International 10-20 electrode placement according to the guidelines by the International Federation of Clinical Neurophysiology (IFCN).

Case B. Case B. The patient's EEG was recorded using the Natus EEG System (Natus Medical) with the International 10-20 electrode placement according to the guidelines by the International Federation of Clinical Neurophysiology (IFCN).

Genetic testing and data analysis

Case A. Exome sequencing was performed in SCN1A, with the proband and the two unaffected parents. The process involved DNA extraction and sonication (Covaris, Woburn, USA), followed by library preparation using the Medexome kit (Roche, Meylan, France) as per the manufacturer's instructions. Paired-end 2 × 75 sequencing was carried out on a NextSeq500 instrument (Illumina, San Diego, USA). Genomic alignment against the hg19/GRCh37 assembly and variant calling were, respectively, done with BWA-MEM v.0.7.12(1) and GATK HaplotypeCaller v.3.4 (Broad Institute, Boston, MA, USA) while QC were evaluated using DeCovA(2). Only highly-confident variants were kept for analysis (total depth >9; alternative allele depth >4; no strand bias; mosaicism >10%). Rare variants were considered as having a frequency below 1% in the gnomAD database.

Case B was diagnosed by a commercial epilepsy gene panel via Invitae.

All pertinent inheritance patterns were considered. All identified variants were evaluated with respect to their pathogenicity and causality. Variants were categorized into five classes (pathogenic, likely pathogenic, variant of uncertain significance (VUS), likely benign, and benign) according to the American College of Medical Genetics (ACMG) guidelines(3).

Online *in silico* analysis tools were used to assess how deleterious the discovered substitutions are. These tools included the MutationTaster(4), Sorting Intolerant From Tolerant (SIFT)(5), Polymorphism Phenotyping v2 (PolyPhen-2)(6), Rare Exome Variant Ensemble Learner (Revel)(7), Protein Variant Effect Analyzer (Provean)(8) and Combined Annotation Dependent Depletion (CADD)(9), and Alphamissense(10).

Analysis of the variants was performed using algorithms for computational scoring of splice sites based on different concepts using default parameter settings: Neural Network Splice Prediction, MaxEntScan, Splice site Finder Like, and GeneSplicer available through Alamut Visual V.3.15 (Interactive Biosoftware, France) and splice AI ®[29] through Splice AI visual representation(11) available on Mobidetail application(10).

Minigene splicing reporter assay

A 558-bp *SLC12A5* fragment (the 232 last bases of intron 1, 95 bases of exon 2, the 231 bases of intron 2) was amplified from control DNA by HotStarTaq DNA Polymerase kit (Qiagen®) using the forward primer 5′-CTAAACAGCCACATATGCCAAGAGCAAGGAAGCACTC-3′ and reverse primer 5′-CCCCCCTCGACCATATGGAGGAACAGGTGAGGAGAACAG-3′. Amplicon was inserted (In-Fusion HD Cloning Kit, Ozyme) into the *Nde*I restriction site of the previously described pTB2 minigene vector. Minigene mutant constructs were generated by site-directed mutagenesis. Normal and mutant minigenes were transfected, in triplicate, in HeLa cells with the Fugene HD transfection reagent kit (Promega) to analyze spliced products.

After total RNA extraction with Kit RNAqueous-4PCR (Ambion) and reverse transcription with Transcriptor High Fidelity cDNA Synthesis Sample kit (Roche), cDNA from normal and mutant transfections were amplified by HotStarTaq DNA Polymerase (Qiagen) using the forward primer 5′-CAACTTCAAGCTCCTAAGCCACTG-3′ and reverse primer 5′-GGTCACCAGGAAGTTGGTTAAATCA -3′. The RT–PCR products were studied by agarose gel electrophoresis (2%) and sequenced on the ABI 3500 Dx Genetic Analyzer (Applied Biosystems) using BigDye™ Terminator v3.1 Cycle Sequencing Kit (Applied Biosystems).

Structural analysis of KCC2

The authors have used the Protein Data Base entry 623m for the structural analysis. A structural optimization of Arg[420] has been performed with a single round of real space refinement in Coot(12) and structural depictions have been generated with CCP4mg(13).

Expression constructs

The KCC2 in the homemade vector including ubiquitin promoter (14) was created by synthesis of the insert encoding the coding region of human KCC2b isoform (NM_020708.4) and subcloning into the vector using *NHE*1 and *EcoR*1 restriction sites. Thereafter, this construct, denominated $KCC2_{WT}$ (wild-type), was used as a backbone to create constructs encoding

KCC2$_{F117I}$ and KCC2$_{R420C}$ variants and the intracellular C-terminus domain (KCC2$_{CTD}$). KCC2$_{F117I}$ and KCC2$_{R420C}$ variants were created by insertion of mutations Phe117Ile and Arg420Cys, respectively. The KCC2$_{CTD}$ encoding KCC2's amino acids 637 through 1116 was created by deletion first 636 amino acids. The synthesis or mutagenesis of all constructs was performed by GenScript.com. The construct encoding pH-Sensor was created by subcloning the ecliptic pHluorin (AF058695) into the p-mCherryC1 vector (Clontech). The sequence of the linker was LRSRAQASNSAVDT. The construct encoding pEYFP-Mem, a fusion protein consisting of the N-terminal 20 amino acids of neuromodulin, and an enhanced yellow fluorescent protein EYFP was from Clontech. The neuromodulin fragment contains a signal for posttranslational palmitoylation of cysteines 3 and 4 that targets EYFP to membranes. The construct encoding Homer1b-eGFP was a gift from Dr. Laurent Fagni. All constructs were verified by DNA sequencing. The sequences of the constructs as well as the cDNAs are available on request.

Chemicals.

Unless otherwise specified, all cell culture reagents were obtained from Thermo Fisher Scientific (Waltham, MA, USA). General laboratory chemicals were purchased from Sigma-Aldrich (St. Louis, MO, USA). Ion channel and transporter blockers were obtained from Tocris Bioscience (Bristol, UK). All products were purchased through the respective French or German distributors.

Neuronal and Cell line cultures. Transfection.

Human embryo kidney cells (HEK293) and Mouse neuroblastoma cells (N2a) were acquired from ATCC (CRL-1573™ and CCL-131, respectively). Both cell types were cultured in 50% minimal essential medium (MEM), 50% Dulbecco's modified Eagle's medium (DMEM) supplemented with 8% FBS and 10 IU/ml penicillin/streptomycin. The cells were transfected with appropriate pcDNAs using Lipofectamine 2000 (Invitrogen) or TurboFect (Thermo Fisher Scientific) according to the manufacturer's protocol and used 36-48 h after transfection.

The primary cultures of 18 days rat embryo hippocampi were prepared and transfected with the indicated cDNA constructs as described previously (15).

To visualize the neurons expressing exogenous KCC2 variants, we co-transfected them with cDNA encoding eGFP (for patch clamp recordings) or Homer1b-eGFP for analysis of spines

density or pEYFP-Mem for study of the surface expression. KCC2/eGFP, KCC2/Homer1b-eGFP or KCC2/pEYFP-Mem cDNA ratio was 5:1 (w/w). We showed previously that at the mentioned ratio, all neurons expressing eGFP are co-expressing ectopic KCC2 (Fig.5 E) (14).

Patch-clamp recordings.

Coverslips containing transfected neurons were transferred to the stage of an inverted microscope and perfused with extracellular solution containing (in mM): 140 NaCl, 2.5 KCl, 20 HEPES, 20 D-glucose, 2 $CaCl_2$, and 2 $MgCl_2$ (pH 7.4). The solution was supplemented with 10 μM bumetanide and 1 μM tetrodotoxin (TTX).

Drugs were applied using a multibarrel perfusion system positioned ~250 μm from the soma to ensure rapid solution exchange.

Recordings were performed in voltage-clamp mode using a MultiClamp 700B amplifier and pCLAMP software (Axon Instruments). Signals were filtered at 2 kHz and digitized at 10 kHz. Input resistance (Rin) and membrane capacitance were determined from responses to ±10 mV voltage steps applied from a holding potential of −70 mV.

All recordings were performed at 22°C from pyramidal-like neurons expressing eGFP.

Soma-dendrite gradient Cl^- extrusion assay

KCC2-mediated Cl^- transport in cultured neurons was assessed using a somato-dendritic Cl^- gradient assay as previously described by Jarolimek et al., (1999) and Kelsch et al., (2001) (16,17).

The assay involves whole-cell patch-clamp measurement of the reversal potential of $GABA_A$ receptor-mediated current responses (E_{GABA}) at the soma and dendrites, followed by calculation of the somato-dendritic Cl^- gradient. This method has been validated as an effective measure of KCC2-mediated Cl^- extrusion capacity (18–20).

Neurons were transfected at 3 days *in vitro* (DIV) and recorded at 6-8 DIV. E_{GABA} was determined during brief (50–100 ms) focal applications of 20 μM isoguvacine ($GABA_A$ receptor agonist) applied to the soma and to a primary dendrite 50–60 μm from the soma. Isoguvacine was dissolved in extracellular solution and delivered via a micropipette connected to a Picospritzer (General Valve Corporation; 5 psi). Patch electrodes (6.5–7.5 MΩ) were filled with intracellular solution containing (in mM): 29 KCl, 130 K-gluconate, 10 HEPES, 1.1 EGTA, 0.1 $CaCl_2$, 4 MgATP, and 0.3 NaGTP (pH 7.2; 280 mOsm). Membrane potential was held at −70 mV. All voltages were corrected for a 14.5 mV liquid junction potential. After

achieving whole-cell configuration, recordings began following a 5-min equilibration period. E_{GABA} was calculated from current–voltage (I–V) relationships obtained by measuring peak isoguvacine responses at −79, −59, −39, and −19 mV. Intervals between applications were 5 s.

Gramicidin perforated patch Clamp recording

Gramicidin perforated patch-clamp recordings were performed as previously described (21). Patch pipettes (5–6 MΩ) were filled with solution containing (in mM): 150 KCl and 10 HEPES, supplemented with gramicidin A (20 µg/ml), pH 7.2. Isoguvacine (20 µM) was applied to the soma and proximal dendrites using a Picospritzer. A test pulse at −60 mV was used to determine the direction of I_{GABA} and optimize agonist application to obtain brief (300–1000 ms) responses with amplitudes < ±100 pA. Depending on current polarity at −60 mV, I–V relationships were obtained at: -116, -96, -76, -56 mV (for outward currents or -76, -56, -36 and -16 mV (for inward currents. All voltages were corrected for a -4 mV liquid junction potential.

mEPSCs recordings

Miniature excitatory postsynaptic currents (mEPSCs) were recorded in the presence of 10 µM bicuculline to block $GABA_A$ receptor-mediated currents. The intracellular solution contained (in mM): 120 CsCl, 10 HEPES, 1.1 EGTA, 0.1 $CaCl_2$, 10 sodium phosphocreatine, 4 MgATP, and 0.3 NaGTP (pH 7.2; 280 mOsm). Patch pipette resistance was 5–6 MΩ. Neurons were transfected at 6 DIV and recorded at 14–16 DIV. mEPSCs were analyzed using Mini Analysis software (Synaptosoft, Decatur, GA, USA).

Western blot analysis

Transfected HEK293 cells were lysed in buffer containing 50 mM tris-HCl (pH 7.5), 1 mM EGTA, 1 mM EDTA, 50 mM sodium fluoride, 5 mM sodium pyrophosphate, 1 mM sodium orthovanadate, 1% (w/v) Triton X-100, 0.27 M sucrose, 10mM dithiothreitol (DTT), and protease inhibitors (complete protease inhibitor cocktail tablets, Roche, 1 tablet per 50 ml) followed by centrifugation. All cleared lysates were diluted in SDS sample buffer (1% BME, 2% SDS, 0.05 mg/mL bromophenol blue, 65 mM Tris, pH 6.8), heated at 37°C for 10 min, and analyzed using 4-12% Tris-glycine gradient gels (Invitrogen).

The antibodies used for protein detection were: chicken anti-KCC2 polyclonal primary antibody, generously provided by Claudio Rivera, dilution 1:4000; Rabbit KCC2 (Phospho-Thr1007) Polyclonal Antibody (WP-29292, 1:4000, Bertin technologies); Rabbit KCC2 (Phospho-Ser940) Polyclonal Antibody (TA309219, 1:2000, Origene); Beta-actin mouse monoclonal antibody (MA515739, 1:8000, Thermo Fisher Scientific). The secondary

antibodies were goat anti-Rabbit IgG, goat anti-mouse IgG or goat anti-chicken IgY, all coupled to horseradish peroxidase and used in 1:10000 dilution (Thermo Fisher Scientific, #32260, # 31430; #A16054, respectively). Signals were detected using a G:BOX Chemi XT4 imaging system (Syngene). The relative intensities of immunoblot bands were determined by densitometry with Metamorph software (Molecular Devices).

Antibodies for immunocytochemistry

Primary antibodies used for immunocytochemistry were rabbit polyclonal anti-KCC2 N1-12; 1:1000 (Neuromab, California, USA) and chicken polyclonal anti-GFP antibody # 600-901-215; 1:400 (ThermoFisher Scientific). Secondary antibodies were Cy3-conjugated goat anti-rabbit IgG (dilution 1:400; Jackson ImmunoResearch Laboratories), Alexa Fluor 488–conjugated goat anti-chicken IgG (dilution 1:300; FluoProbes).

Tl^+ flux assay

Transport activity of hKCC2 was determined by Cl^--dependent uptake of Tl^+ in HEK-293 cells as described previously (22,23). To initiate the flux measurement, the medium in the 96-well culture dish was replaced by 80 µl hypotonic preincubation buffer (100 mM N-methyl-D-glucamine-chloride, 5 mM Hepes, 5 mM KCl, 2 mM $CaCl_2$, 0.8 mM $MgSO_4$, 5 mM glucose, pH 7.4; osmolarity: 175 mmol/kg ± 2) with 2 µM FluoZin-2 AM dye (Invitrogen) plus 0.2% (wt/vol) Pluronic F-127 (Invitrogen) and incubated for 48 min at RT. Afterward, cells were washed three times with 80 µl preincubation buffer and incubated for 15 minutes with 80 ml preincubation buffer including 0.1 mM ouabain to block the activity of the Na^+/K^+ ATPase. Then, the 96-well plate was placed into a fluorometer (Fluoroskan FL, Thermo Scientific), and each well was injected with 40 µl 5x Thallium stimulation buffer (12 mM Tl_2SO_4, 100 mM N-methyl-D-glucamine 5mM Hepes, 2 mM KCl, 2 mM $CaCl_2$, 0.8 mM $MgSO_4$, 5 mM glucose, pH 7.4). The fluorescence was measured in a kinetic-dependent manner (excitation 485 nm, emission 538 nm, one frame in 6 s in a 200-speriod) across the entire cell population in a single well. By using linear regression of the initial values of the slope of Tl^+- stimulated fluorescence increase, the transport activity was calculated. The absolute values are normalized by setting the slope of the $hKCC2^{WT}$ as 100 % and calculating the percentage of the activity of the mutants respectively.

$NH4^+$ flux assay

NH_4^+-induced pH_i shifts described previously (24,25) were used to monitor KCC2-dependent pH_i changes in N2a cells co-transfected with pH-Sensor (26) and one or two cDNA constructs encoding KCC2 variants. The acquisition of fluorescence images was performed using a customized fluorescence set-up and consecutive cells imaging using the following pairs of excitation (ex.) and emission (em.) filters: ex. 490/10; em. 535/50 (pH sensitive channel), ex.577/25; em. 632/60 (pH insensitive). Images were obtained every 10s (0.1 Hz) with a 40x objective.

The duration of excitation was selected for each cell type to avoid use-dependent bleaching of the signal. Individual coverslips were transferred to a specially designed recording chamber where they were fully submerged and superfused at room temperature with HEPES-buffered saline (HBS) containing 140 mM NaCl, 2.5 mM KCl, 20 mM Hepes, 20 mM D-glucose, 2.0 mM CaCl2, 2.0 mM MgCl2, (pH 7.4). 10 µM bumetanide was added routinely to all solutions in order to prevent the potential contribution of NKCC1 to the change of pH_i during NH_4^+ application. Each experiment started from 5 min of pH_i baseline recordings followed by 6 to 8 min applications of HBS containing 10 mM of NH_4Cl with consecutive washes of NH_4Cl for another 5 min. The offline creation and analysis of the ratiometric images (**Δ**F/F0) was performed using Metamorph software (Roper Scientific SAS, Evry, France) as described previously(26).

Surface expression analysis

For analysis of the surface expression of KCC2 variants, the images of N2A cells, co-transfected with pEYFP-Mem and one of the KCC2 variants or $KCC2_{CTD}$, were acquired using a Zeiss LSM 710 equipped with an Airyscan module (allowing a resolution around 150 nm) and a plan Apo 63X/1.40 oil objective. Five planes separated by 0.185µm were acquired in Airyscan mode and processed for reconstruction with a strength of 8. A maximal projection was used for analysis.

All further image treatments were done using imageJ software. For quantification of signal on the membrane and cytoplasm, images of cells were segmented using the membrane staining of pEYFP-Mem, from which the whole cell and cytoplasm were defined. On the created mask, the touching cells were separated manually. These masks were used to measure the mean intensity

signal of KCC2 in the membrane and in the cytoplasm from the KCC2 channel after background subtraction. All measurements were done blind to the KCC2 plasmid construct.

Statistical analysis

Statistical analyses and assessment of normal distribution (Shapiro-Wilk test) were performed with GraphPad Prism 10 (GraphPad). For data showing normal distribution and with n>19, one-way ANOVA and the post hoc Tukey test were used for multiple comparisons between groups. For non-matched data showing a non-normal distribution or involving a low number of experiments ($n < 20$), the Kruskal–Wallis test followed by Dunn's post hoc test was used to compare three or more independent groups. For matched or paired data (e.g., western blots), the Friedman test with Dunn's multiple comparisons post hoc test was applied. All values reported in the text are presented as mean ± SD. In both the text and figure legends, *N* denotes the number of independent experiments, while *n* refers to the number of recorded cells, wells, or western blot membranes used for analysis (*N; n*).

For the boxplots, the box extends from the first (Q1) to the third (Q3) quartiles. The line inside the box represents the median, and the "+" symbol denotes the mean. The whiskers indicate the minimum and maximum values. Statistical significance was considered at $P < 0.05$. Asterisks indicate the range of *P* values: *$P < 0.05$; **P<0.01; ***P<0.001 and ****P<0.0001. Details on the number of experiments, statistical tests, Q and P values are provided in the Figure Legends and Supplementary Tables S4–S8.

Supplementary Results

Antiseizure treatment.

Case A. Multiple antiseizure medications failed to control seizures. They included: valproate, vigabatrin, carbamazepine, clobazam, clonazepam, topiramate, zonizamide, phenobarbital, phenytoin, carbamazepine, benzodiazepine, levetiracetam, rufinamide, cannabidiol, eslicarabazepine, stiripentol, bromide, lacosamide, and perampanel. Vagus nerve stimulation and the ketogenic diet were also ineffective.

Bumetanide, a diuretic, has been investigated for its potential to alleviate autism spectrum disorder symptoms(27), tried twice and was ineffective too.

Case B. Multiple antiseizure medications were administered, including levetiracetam, phenobarbital, clobazam, carbamazepine, lacosamide, and continuous midazolam infusion, without adequate seizure control. Introduction of a classic ketogenic diet during the third week of life, in combination with levetiracetam, phenobarbital, and clobazam, resulted in a partial reduction in seizure frequency, duration, and intensity.

***In-silico* analysis:**

Case A. *In-silico* prediction scores with SpliceAI were in favor of highly deleterious effects for case A variants: CADD=26.3, Alphamissense=1, and REVEL=0.965 for variant NG_046341.2:g.19162T>A, CADD=31, AlphaMissense=0.998, and REVEL=0.821 for variant NG_046341.2:g.26969C>T. g.19162T>A was absent from the population databases gnomAD_V4.1 and AllofUS, and g.26969C>T was only reported once, at the heterozygous state.

Case B. *In silico* splice prediction analysis of the *SLC12A5* variant NG_046341.2:g.18262A>C. The splice-disrupting effects of the *SLC12A5* variant NG_046341.2:g.18262A>C were evaluated using *in silico* splice prediction tools. This variant affects a canonical splice acceptor site shared by multiple protein-coding transcripts, including NM_001134771.2:c.122-2A>C and the MANE Select transcript NM_020708.5:c.53-2A>C.

SpliceAI predictions were identical across transcripts, reflecting disruption of the same genomic splice acceptor site. For NM_001134771.2: c.122-2A>C (Supplementary Table S1), SpliceAI predicted a high-confidence loss of the canonical splice acceptor site (Δ score = 0.99) located 2 bp from the variant. Additional lower-confidence predictions included the creation of a cryptic upstream splice acceptor site 23 bp from the variant and weak donor loss. Pangolin independently predicted splice site loss at the same acceptor position (Δ score = 0.86), supporting a deleterious effect on pre-mRNA splicing. All predictions were generated on the plus strand (GRCh38).

Consistent results were obtained for the MANE Select transcript NM_020708.5:c.53-2A>C (Supplementary Table S2). SpliceAI predicted near-complete loss of the canonical splice acceptor site (Δ score = 0.99), together with a potential cryptic upstream acceptor site (Δ score = 0.61 at −23 bp) and weak donor loss (Δ score = 0.10). Pangolin also predicted splice site loss (Δ score = 0.86) and a possible upstream splice gain (Δ score = 0.47). The variant showed a high CADD score (35), further supporting its functional impact.

Structural modeling

As indicated by the structural determination, KCC2b functions as a homodimer, visualized with monomeric subunits shown in distinct colors (**Fig.2D**).

Variant Phe117Ile: This substitution produces no apparent structural rearrangements. Both residues are hydrophobic, and the replacement of the bulkier Phe117 with the smaller Ile117 does not introduce steric clashes (**Fig. 2D, d.1–d.2**). However, the residue lies in close proximity to the K^+-binding site, where even minor alterations could subtly influence the ion transport mechanism.

Variant Arg420Cys: In the wild-type protein, the side chain of Arg420 forms an extended hydrogen bond network with the backbone oxygens of Leu71 and Thr69 in the N-terminus (**Fig. 2D, d.3**)(28). These interactions stabilize an autoinhibitory loop that seals the transporter entrance in the inward-open conformation, preventing ion translocation. In the Arg420Cys variant, substitution of Arg420 with Cys420 disrupts this network (**Fig. 2D, d.4**), potentially releasing the N-terminus and promoting transporter activation.

Variant Leu766Arg: This substitution is located within the intracellular C-terminal domain, adjacent to a hydrophobic patch (**Fig. 2D, d.5–d.6**). Structural modeling predicts that the Leu→Arg exchange alters the surface electrostatic potential, converting a hydrophobic region into a positively charged surface, which could influence protein–protein interactions or regulatory dynamics within the *C*-terminus. Changes in these interactions can lead to the release of the *N*-terminus and thus activate the transporter.

Supplementary Figures:

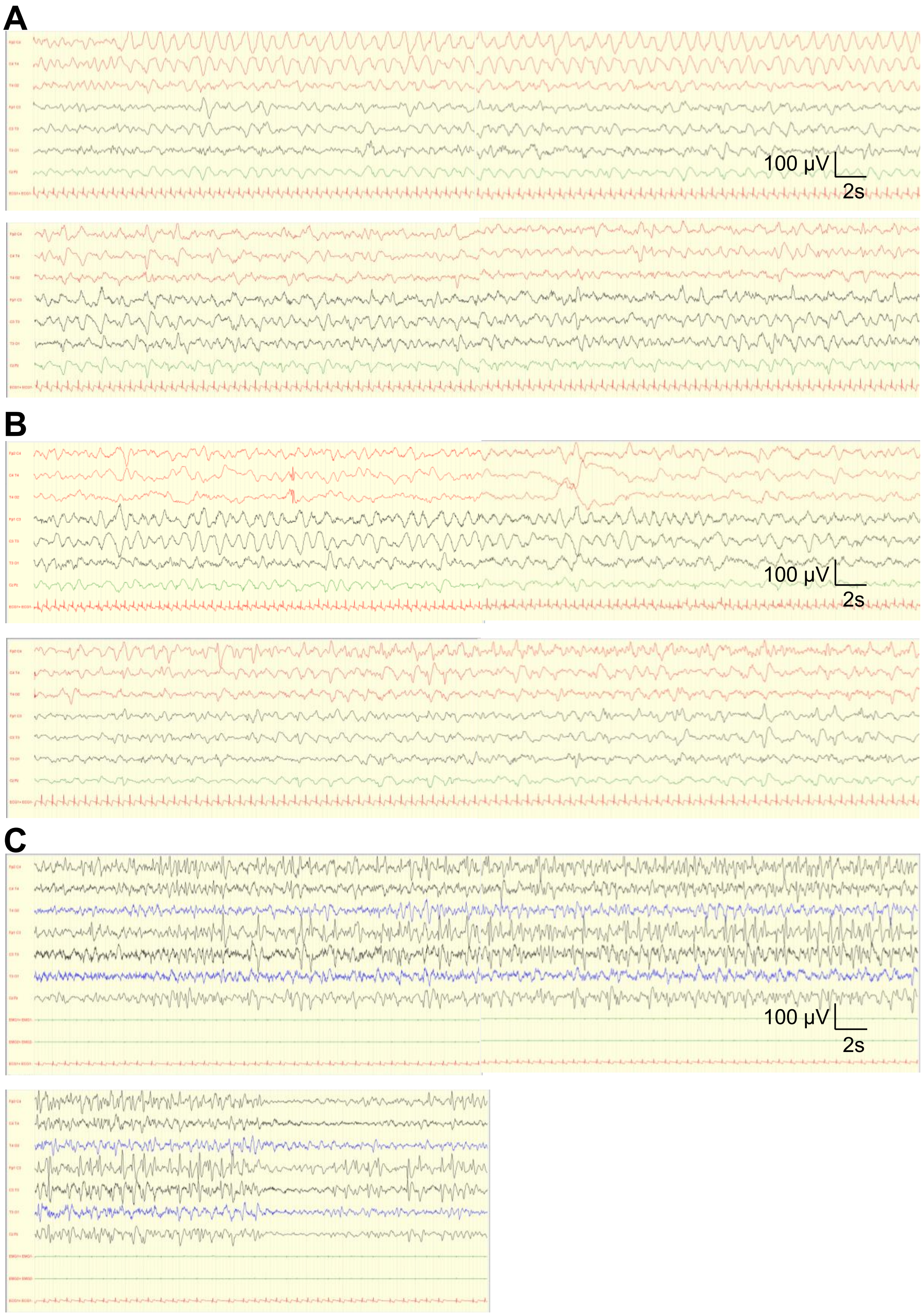

Supplementary Figure S1: Case A. Different types of multifocal seizures recorded at ages 7 months (A and B) and 16 months (C). (continued on the next page)

A. Seizure A (7 months). The upper panel shows generalized clonic jerks involving the head and limbs, accompanied by crying, apnea, and hypertonia, associated with right anterior rhythmic delta activity on EEG. The lower panel shows the EEG recorded 2 minutes later. Clinical evolution includes persistent crying, hypertonia, and deviation of the head to the right. The EEG demonstrates attenuation of the right-sided rhythmic activity, while rhythmic delta activity and spikes become predominant over the left frontocentral region.

B. Seizure B (7 months). The upper panel illustrates generalized clonic jerks with hypertonia, apnea, and cyanosis, associated with left-sided rhythmic delta activity. The lower panel shows the progression of clinical manifestations with crying and deviation of the head and eyes to the right. The EEG demonstrates attenuation of the left-sided activity, with emerging rhythmic delta activity showing right frontocentral predominance.

C. Seizure C (16 months). Subcontinuous subclinical focal discharges are predominant over the frontocentral regions. It is difficult to determine whether this pattern reflects the propagation of a single discharge or two superimposed seizures.

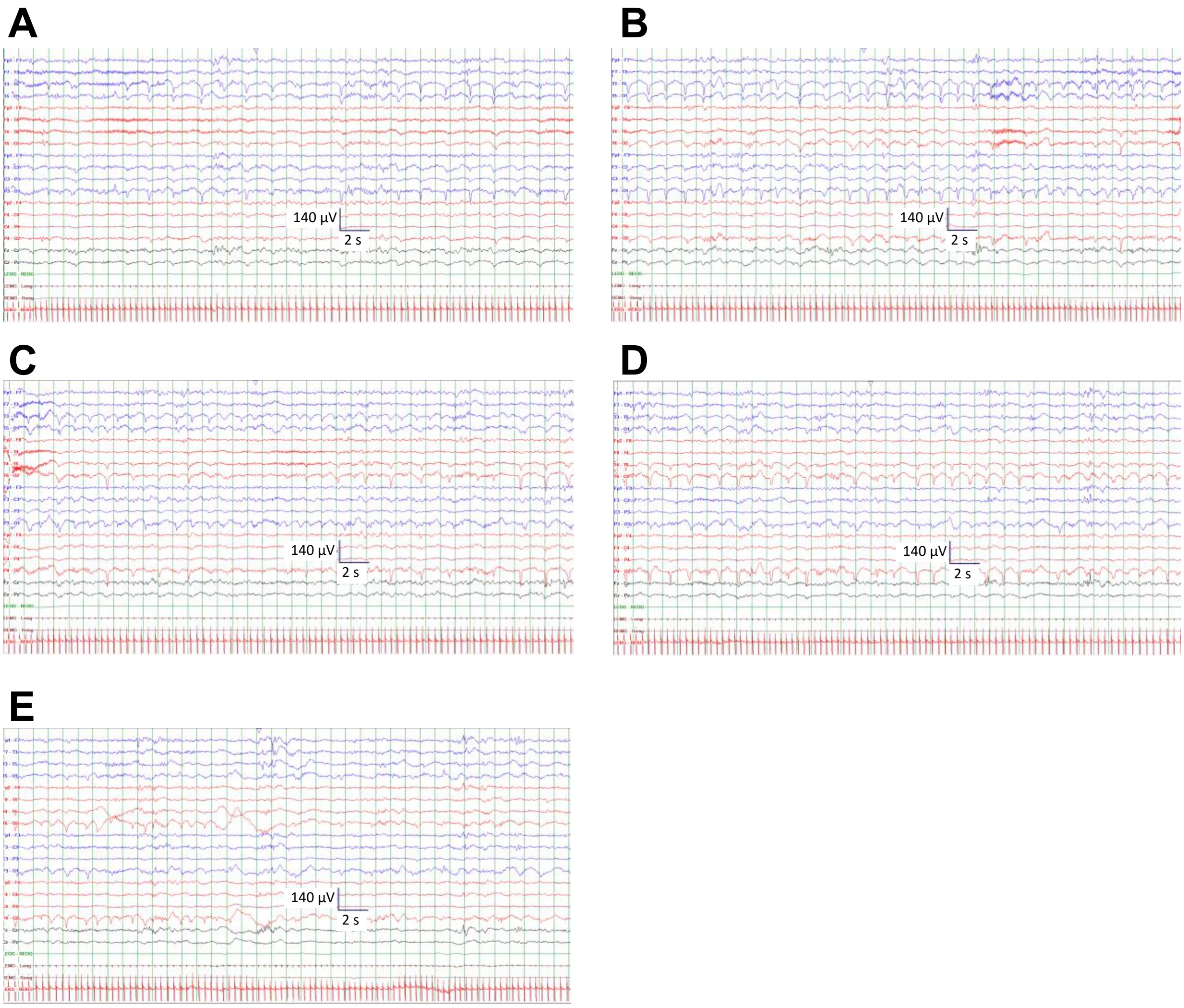

Supplementary Figure S2: Case B. A to E, consecutive EEG recorded during the first week of life.

EEG demonstrates a focal seizure with minimal clinical signs arising from the left posterior quadrant (T5, P3, O1) in A and B. In C, the seizure starts over the right posterior quadrant at T6, P4, O2, and evolves over the posterior quadrant in D, until the seizure ends in E.

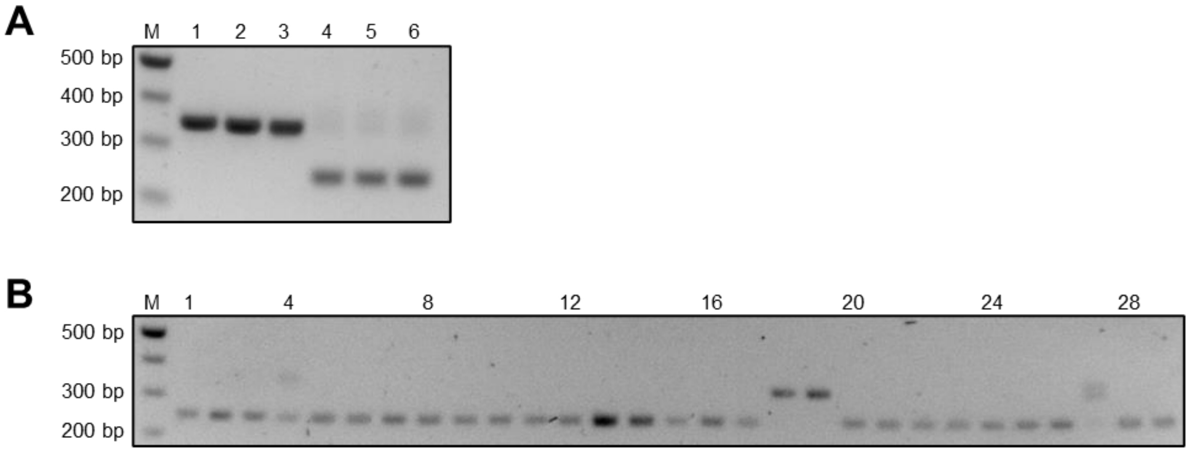

Supplementary Figure S3: Minigene splicing assay for the *SLC12A5* c.53-2A>C.

Ex vivo analysis was performed in HeLa cells transfected with either the wild-type (WT) c.53-2A or the mutant c.53-2A>C minigene (variation identified in exon 2 of the *SLC12A5* gene).

A. Gel electrophoresis of RT-PCR products obtained after transfection of HeLa cells with wild-type (lanes 1 to 3) and mutated (lanes 4 to 6) constructs. Transcripts obtained from the mutated minigene were shorter than those from the WT minigene, suggesting exon skipping. The effect is not entirely complete as there is still a small amount of transcript from the mutated minigene, which seems to have the same size as transcripts from the normal minigene.

B. TOPO TA cloning: To measure the amount of normal and aberrant transcripts from the mutated minigene, we performed TOPO TA cloning (ThermoFisher) on the cDNA from the mutated minigene. Only 3 of the 29 selected clones were of normal size, while the rest of the clones were shorter, suggesting exon skipping in almost all transcripts under our experimental conditions.

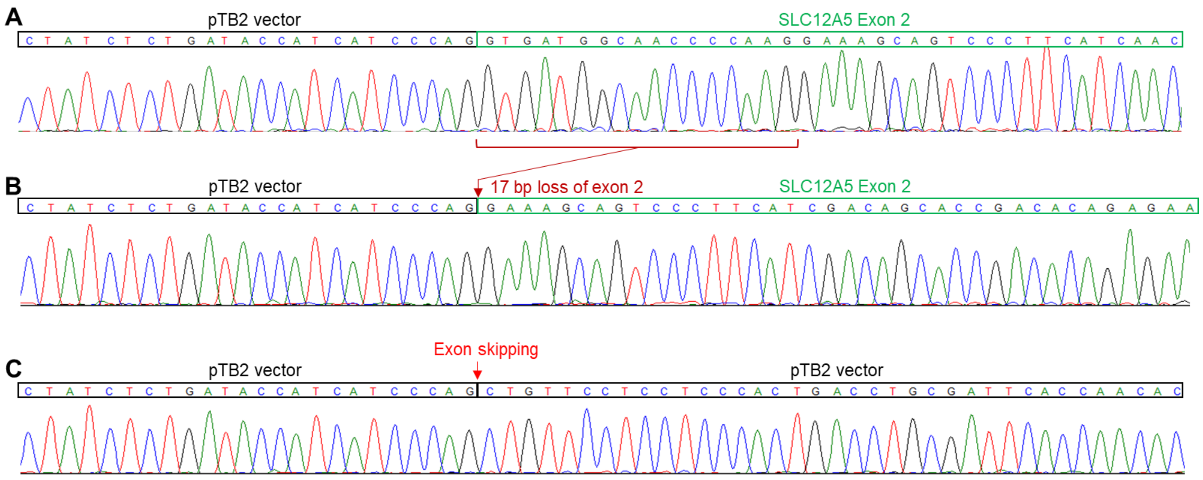

Supplementary Figure S4: RT-PCR and TOPO TA product Sanger sequencing.

A. Sequence of cDNA from RT-PCR from WT minigene transcript: see normal splicing with the presence of exon 2.

B. Sequence of cDNA from B19 TOPO TA clone from mutated minigene transcript: apparently normal splicing product shows a 17 bp deletion of exon 2, leading to a frameshift effect and probably LOF.

C. Sequence of cDNA from B17 TOPO TA clone from mutated minigene transcript: exon 2 skipping.

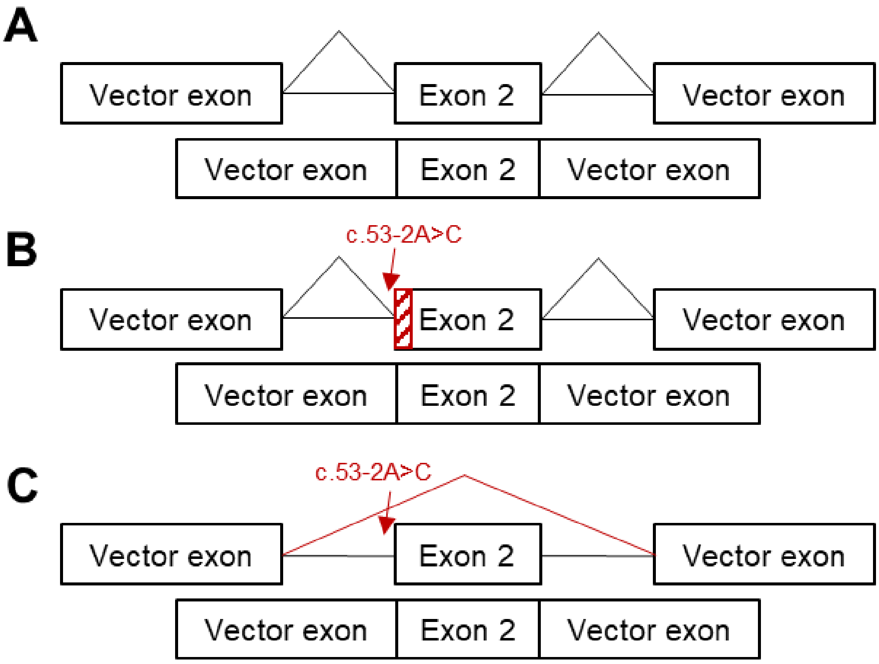

Supplementary Figure S5: Schematic representation of RT-PCR products from minigene experiments in HeLa cells, shown in Supplementary Figure S3.

A. normal minigene.

B. 17pb loss of exon 2 for the mutant minigene.

C. Complete exon skipping.

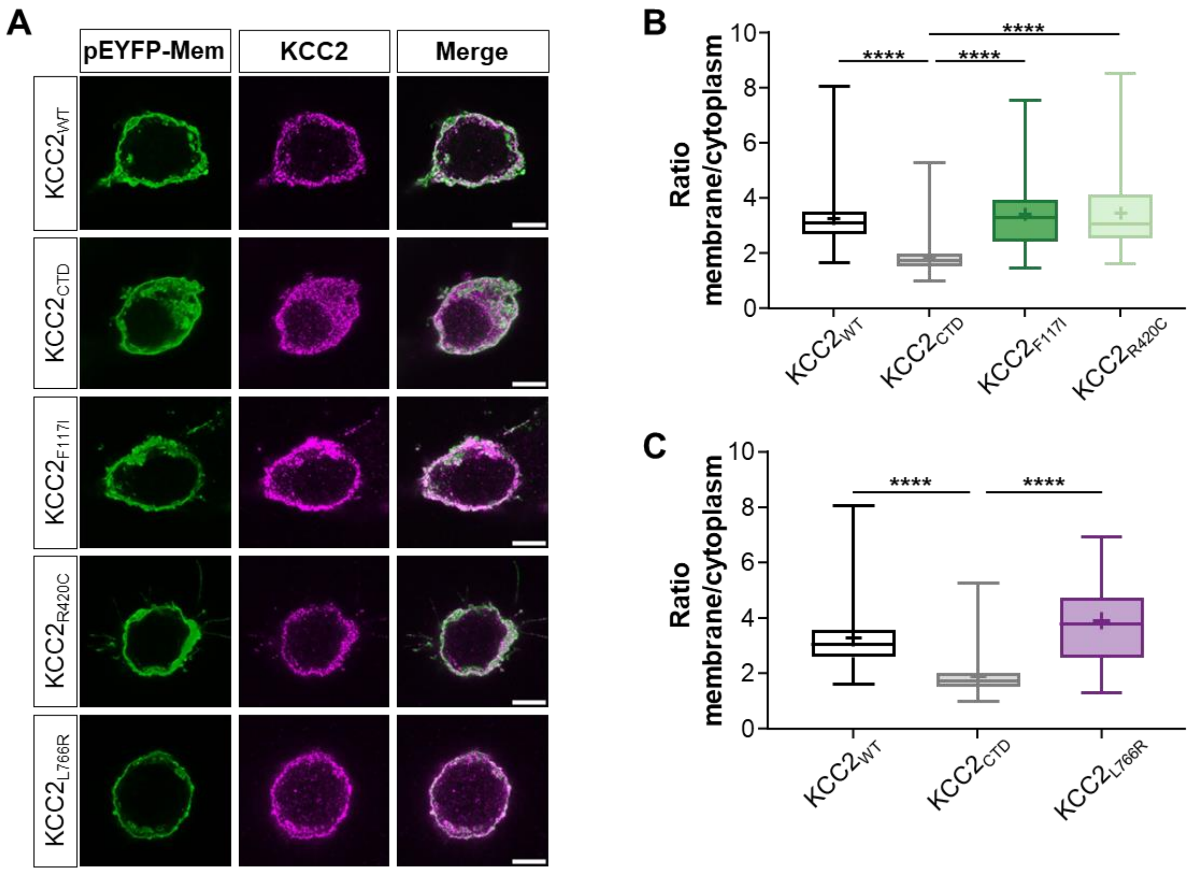

Supplementary Figure S6: Surface expression of different variants of KCC2 co-transfected with a fragment of neuromodulin linked to enhanced yellow-green fluorescent protein (pEYFP-Mem). As negative control cells were transfected with a construct encoding KCC2's carboxyl terminus domain (KCC2$_{CTD}$), which could not be expressed in the plasma membrane due to the lack of hydrophobic transmembrane domains. The analysis of the properties of variant KCC2$_{L766R}$ was performed in a distinct set of experiments that included appropriate positive and negative control conditions. Therefore, the corresponding results are shown in a separate figure, apart from those illustrating the results for variants KCC2$_{F117I}$ and KCC2$_{R420C}$.

A. Representative images of immunofluorescence using confocal Airy scan mode that show pEYFP-Mem (green) and KCC2 (magenta) in N2a cells expressing the indicated constructs. Scale bar: 5µm.

B-C. Boxplots represent the quantification of the plasma membrane/cytoplasm ratio. In B: N=4; n=58 for KCC2$_{WT}$, n=66 for mock, n=68 for KCC2$_{F117I}$, n=64 for KCC2$_{R420C}$. In C: N=3; n=40 for KCC2$_{WT}$, n=46 for mock, n=38 for KCC2$_{L766R}$. Compared using the Kruskal-Wallis test followed by Dunn's post hoc test.

See **Supplementary Table S8** for exact values and statistical comparisons.

Supplementary Tables:

Supplementary Table S1. Clinical Characteristics of Patients with Pathogenic *SLC12A5* Variants.

This table summarizes the phenotypic and genotypic features of the patients included in the current study (Cases A and B) and compares them with previously reported cases in the literature. References are indicated numerically as follows: [1]–[4].

[1], Stödberg et al., 2015 https://doi.org/10.1038/ncomms9038
[2], Saitsu et al., 2016; https://doi.org/10.1038/srep30072
[3], Saito et al., 2017; https://doi.org/10.1111/cge.13049
[4], Järvelä et al., 2024; https://doi.org/10.3389/fnmol.2024.1372662

n indicates the number of affected individuals. A plus sign (+) indicates that all reported individuals in the referenced study were affected for the specified feature.

Abbreviations: F, female; M, male; AR, autosomal recessive; h, hours; d, days; mo, months; y, years; EEG, electroencephalography; MRI, magnetic resonance imaging; EIMFS, epilepsy of infancy with migrating focal seizures.

Features		Case A	Case B	Previous reports n, [ref]
**Gender**	F	-	-	1, [1]; 3, [2]; 1,[4]
	M	+	+	3, [1]; 1, [2]; 1,[3]
**Inheritance**	AR Compound heterozygous	+	+	2, [1]; 4, [2]; 1, [3]
	AR Compound homozygous	-	-	2, [1]; 1,[4]
**Type of variant**	Missense	+	+	4, [1]; 4, [2]; 2, [3]; 2, [4]
	splice variants	-	+	1, [2]
	deletion	-	-	1, [2]
**Diagnosis**	EIMFS	+	+	+
**Age of onset of seizures**	0-24h	+	+	1, [4]
	1-7d	-	-	3, [3]
	1-6mo	-	-	4,[1]; 1, [2]; 1, [3]
**Seizure type**	Tonic	+	+	+
	Clonic	-	-	+, [2]; +, [4]
	Tonic-clonic	-	-	+, [2]; +, [3]; +, [4]
	Atonic	-	-	+, [1]
**EEG features**	Slow background	-	-	+, [1]; +, [2]
	Discontinuous background	-	+	-
	Multifocal epileptiform discharges	+	+	+
**Response to treatment**	Medically refractory	+	+	+, [1]; 1, [2]; +, [4]
	Pharmacoresponsive	-	-	3, [2]; +, [3]
**MRI findings**	Delayed myelination	-	-	3, [1]; 3, [2]; 1, [4]
	Atrophy	+	-	3, [1]; 4, [2]; 1, [3]
	Corpus callosum abnormalities	-	-	2, [2]; 1, [4]

Features		Case A	Case B	Previous reports<br>n, [ref
Comorbidities	Microcephaly	+	-	3, [1]; 4, [2]; 1, [4]
	Muscle tonus: hypertonia	+	-	-
	Muscle tonus: hypotonia	-	+	2, [1]; 4, [2]; 1, [4]
	Motor skills	minimal movement	minimal movement	minimal movement [1]; [2];[4] Slow progression [3]
	Cognitive skills	Profound delay	Profound delay	Porfound delay [1]; [2];[4] Slow progression [3]
Other complications		Bone fractures, generalized stiffness, respiratory fragility	-	Bone fractures, osteopenia, respiratory fragility, cholangitis, cholestasis, [4]
Outcome		Died at 9y	Died at 6mo	Died at 2.5y,1,[1];<br>Died at 4y,1,[4]

Supplementary Table S2. ***In silico*** **splice prediction analysis of the** ***SLC12A5*** **variant NM_020708.5:c.53-2A>C affecting a shared canonical splice acceptor site.**

SpliceAI scores:

Variant	Gene (☐ = MANE Select transcript ☐ = non-coding transcript)	Δ type	Δ score	position
NM_020708.5:c.53-2A>C<br>⇒ 20:46034946 A>C<br>splice acceptor variant<br>UCSC, gnomAD	SLC12A5 (ENSG00000124140.15 / ENST00000243964.7 / NM_020708.5)<br>protein coding MANE Select transcript (plus strand)<br>OMIM, GTEx, gnomAD, ClinGen, Ensembl, Decipher, GeneCards	Acceptor Loss	0.99	2 bp
		Donor Loss	0.10	96 bp
		Acceptor Gain	0.61	-23 bp
		Donor Gain	0.00	

MANE Select Transcript or All Transcripts

Pangolin scores:

Variant	Gene	Δ type	Δ score	position
NM_020708.5:c.53-2A>C<br>⇒ 20:46034946 A>C<br>splice acceptor variant<br>UCSC, gnomAD	SLC12A5 (ENSG00000124140.15 / ENST00000243964.7 / NM_020708.5)<br>protein coding MANE Select transcript (plus strand)<br>OMIM, GTEx, gnomAD, ClinGen, Ensembl, Decipher, GeneCards	Splice Loss	0.86	2 bp
		Splice Gain	0.47	-23 bp

Supplementary Table S3. ***In silico*** **splice prediction analysis of the** ***SLC12A5*** **variant. NM_001134771.2:c.122-2A>C**

SpliceAI scores:

Variant	Gene (☐ = MANE Select transcript ☐ = non-coding transcript)	Δ type	Δ score	position
NM_001134771.2:c.122-2A>C<br>⇒ 20:46034946 A>C<br>splice acceptor variant<br>UCSC, gnomAD	SLC12A5 (ENSG00000124140.15 / ENST00000243964.7 / NM_020708.5)<br>protein coding MANE Select transcript (plus strand)<br>OMIM, GTEx, gnomAD, ClinGen, Ensembl, Decipher, GeneCards	Acceptor Loss	0.99	2 bp
		Donor Loss	0.10	96 bp
		Acceptor Gain	0.61	-23 bp
		Donor Gain	0.00	

MANE Select Transcript or All Transcripts

Pangolin scores:

Variant	Gene	Δ type	Δ score	position
NM_001134771.2:c.122-2A>C<br>⇒ 20:46034946 A>C<br>splice acceptor variant<br>UCSC, gnomAD	SLC12A5 (ENSG00000124140.15 / ENST00000243964.7 / NM_020708.5)<br>protein coding MANE Select transcript (plus strand)<br>OMIM, GTEx, gnomAD, ClinGen, Ensembl, Decipher, GeneCards	Splice Loss	0.86	2 bp
		Splice Gain	0.47	-23 bp

Supplementary Table S4. Summary of Statistical Analyses for Western Blot Data Presented in Figure 3

Data reference	Data structure		*N; n*	Power	Summary
Fig.3 B	Non-normal distribution	Friedman test	7; 14	$Q(3)=15.00$, $P=0.0018$	**
WT vs F117I		post-hoc Dunn's	7; 14	$P=0.32$	ns
WT vs R420C		post-hoc Dunn's	7; 14	$P=0.0023$	**
WT vs L766R		post-hoc Dunn's	7; 14	$P=0.0038$	**
Fig.3 C	Non-normal distribution	Friedman test	4; 6	$Q(3)=9.00$, $P=0.0218$	*
WT vs F117I		post-hoc Dunn's	4; 6	$P>0.9999$	ns
WT vs R420C		post-hoc Dunn's	4; 6	$P=0.0219$	*
WT vs L766R		post-hoc Dunn's	4; 6	$P>0.1325$	ns
Fig.3 D	Non-normal distribution	Friedman test	4; 7	$Q(3)=9.00$, $P=0.0293$	*
WT vs F117I		post-hoc Dunn's	4; 7	$P>0.9999$	ns
WT vs R420C		post-hoc Dunn's	4; 7	$P>0.9999$	ns
WT vs L766R		post-hoc Dunn's	4; 7	$P=0.0219$	*

Supplementary Table S5. Summary of statistical analyses for ion-transport ability data presented in Figure 4

Data reference	Data structure		*N1; n1 vs N2; n2*	Power	Summary
Fig.4 A	Non-normal distribution	Kruskal-Wallis test	4; 58	*Q*(4)=41.06, *P*<0.0001	****
WT vs mock		post-hoc Dunn's	7; 15 vs 7; 15	*P*<0.0001	****
WT vs F117I		post-hoc Dunn's	7; 15 vs 7; 13	*P*=0.0051	**
mock vs F117I		post-hoc Dunn's	7; 15 vs 7; 13	*P*>0.9999	ns
WT vs R420C		post-hoc Dunn's	7; 15 vs 7; 15	*P*>0.9999	ns
mock vs R420C		post-hoc Dunn's	7; 15 vs 7; 15	*P*<0.0001	****
F117I vs R420C		post-hoc Dunn's	7; 13 vs 7; 15	*P*=0.0001	***
Fig.4 B	Non-normal distribution	Kruskal-Wallis test	6; 277	*Q*(4)=128.2, *P*<0.0001	****
WT vs mock		post-hoc Dunn's	6; 107 vs 6; 75	*P*<0.0001	****
WT vs F117I		post-hoc Dunn's	6; 107 vs 6; 42	*P*<0.0001	****
mock vs F117I		post-hoc Dunn's	6; 75 vs 6; 42	*P*>0.9999	ns
WT vs R420C		post-hoc Dunn's	6; 107 vs 6; 53	*P*=0.0005	***
mock vs R420C		post-hoc Dunn's	6; 75 vs 6; 53	*P*<0.0001	****
F117I vs R420C		post-hoc Dunn's	6; 75 vs 6; 42	*P*<0.0001	****
Fig.4 C	Non-normal distribution	Kruskal-Wallis test	4; 64	*Q*(4)=32.82, *P*<0.0001	****
WT vs mock		post-hoc Dunn's	4; 18 vs 4; 13	*P*=0.0004	***
WT vs F117I		post-hoc Dunn's	4; 18 vs 4; 13	*P*=0.01	*
mock vs F117I		post-hoc Dunn's	4; 13 vs 4; 13	*P*>0.9999	ns
WT vs R420C		post-hoc Dunn's	4; 18 vs 4; 20	*P*>0.9999	ns
mock vs R420C		post-hoc Dunn's	4; 13 vs 4; 20	*P*<0.0001	****
F117I vs R420C		post-hoc Dunn's	4; 13 vs 4; 20	*P*=0.0005	***
Fig.4 D	Non-normal distribution	Kruskal-Wallis test	4; 27	*Q*(4)=19.68, *P*=0.0002	***
WT vs mock		post-hoc Dunn's	4; 7 vs 4; 7	*P*=0.0004	***
WT vs F117I		post-hoc Dunn's	4; 7 vs 4; 6	*P*=0.0328	*
mock vs F117I		post-hoc Dunn's	4; 7 vs 4; 6	*P*>0.9999	ns
WT vs R420C		post-hoc Dunn's	4; 7 vs 4; 7	*P*>0.9999	ns
mock vs R420C		post-hoc Dunn's	4; 7 vs 4; 7	*P*=0.0033	**
F117I vs R420C		post-hoc Dunn's	4; 6 vs 4; 7	*P*=0.0143	*
Fig.4 E	Non-normal distribution	Kruskal-Wallis test	4; 48	*Q*(3)=37.56, *P*<0.0001	****
WT vs mock		post-hoc Dunn's	4; 12 vs 4; 12	*P*<0.0001	****
WT vs L766R		post-hoc Dunn's	4; 12 vs 4; 24	*P*=0.0026	**
mock vs L766R		post-hoc Dunn's	4; 12 vs 4; 24	*P*=0.0006	***
Fig.4 F	Non-normal distribution	Kruskal-Wallis test	4; 183	*Q*(3)=82.22, *P*<0.0001	****
WT vs mock		post-hoc Dunn's	4; 60 vs 4; 47	*P*<0.0001	****
WT vs L766R		post-hoc Dunn's	4; 60 vs 4; 76	*P*<0.0001	****
mock vs L766R		post-hoc Dunn's	4; 47 vs 4; 76	*P*<0.0001	****

Supplementary Table S6. Summary of Statistical Analyses of mEPSC Parameters Presented in Figure 5.

Data reference	Data structure		*N1; n1 vs N2; n2*	Power	Summary
Fig.5 C	Non-normal distribution	Kruskal-Wallis test	8; 55	$Q(3)=14.27$, $P=0.0008$	***
WT vs F117I		post-hoc Dunn's	8; 19 vs 8; 20	$P=0.0147$	*
WT vs R420C		post-hoc Dunn's	8; 19 vs 8; 16	$P=0.0011$	**
F117I vs R420C		post-hoc Dunn's	8; 20 vs 8; 16	$P>0.9999$	ns
Fig.5 D	Non-normal distribution	Kruskal-Wallis test	8; 92	$P=0.1906$	ns
Fig.5 G	Non-normal distribution	Kruskal-Wallis test	5; 293	$Q(3)=20.04$, $P<0.0001$	****
WT vs F117I		post-hoc Dunn's	5; 116 vs 5; 62	$P=0.5088$	ns
WT vs R420C		post-hoc Dunn's	5; 116 vs 5; 120	$P=0.0001$	****
F117I vs R420C		post-hoc Dunn's	5; 62 vs 5; 120	$P=0.576$	ns

Supplementary Table S7. Summary of statistical analyses for results from cells co-expressing the Phe117Ile and Arg420Cys variants in Figure 6.

Data reference	Data structure		*N1; n1 vs N2; n2*	Power	Summary
Fig.6 A	Non-normal distribution	Kruskal-Wallis test	3; 15	$Q(4)=9.38$, $P=0.0029$	**
WT vs mock		post-hoc Dunn's	5; 5 vs 5; 5	$P<0.0267$	*
WT vs F117I+R420C		post-hoc Dunn's	5; 5 vs 5; 5	$P>0.9999$	ns
mock vs F117I+R420C		post-hoc Dunn's	5; 5 vs 5; 5	$P<0.0216$	*
Fig.6 B	Non-normal distribution	Kruskal-Wallis test	6; 218	$Q(3)=82.83$, $P<0.0001$	****
WT vs mock		post-hoc Dunn's	6; 107 vs 6; 75	$P<0.0001$	****
WT vs F117I+R420C		post-hoc Dunn's	6; 107 vs 6; 36	$P=0.0598$	ns
mock vs F117I+R420C		post-hoc Dunn's	6; 75 vs 6; 36	$P<0.0001$	****
Fig.6 C	Non-normal distribution	Mann-Whitney U-test			
WT vs F117I+R420C			8; 19 vs 8; 14	$U=96$; $P=0.1833$	ns
Fig.6 D	Non-normal distribution	Mann-Whitney U-test			
WT vs F117I+R420C			8; 19 vs 8; 14	$U=128$; $P=0.8645$	ns
Fig.6 E	Non-normal distribution	Mann-Whitney U-test			
WT vs F117I+R420C			5; 116 vs 5; 109	$U=3072$; $P=0.1296$	ns

Supplementary Table S8. Summary of statistical analyses of cell surface expression data presented in Supplementary Figure S6.

Data reference	Data structure		*N1; n1 vs N2; n2*	Power	Summary
Fig. S6 B	Non-normal distribution	Kruskal-Wallis test	4; 256	$Q(4)=108.4$, $P<0.0001$	****
WT vs CTD		post-hoc Dunn's	4; 58 vs 4; 66	$P<0.0001$	****
WT vs F117I		post-hoc Dunn's	4; 58 vs 4; 68	$P>0.9999$	ns
WT vs R420C		post-hoc Dunn's	4; 58 vs 4; 64	$P>0.9999$	ns
CTD vs F117I		post-hoc Dunn's	4; 58 vs 4; 66	$P<0.0001$	****
CTD vs R420C		post-hoc Dunn's	4; 58 vs 4; 66	$P<0.0001$	****
F117I vs R420C		post-hoc Dunn's	4; 58 vs 4; 66	$P>0.9999$	ns
Fig. S6 C	Non-normal distribution	Kruskal-Wallis test	4; 124	$Q(3)=60.94$, $P<0.0001$	****
WT vs CTD		post-hoc Dunn's	4; 40 vs 4; 46	$P<0.0001$	****
WT vs L766R		post-hoc Dunn's	4; 40 vs 4; 38	$P>0.9999$	ns
CTD vs L766R		post-hoc Dunn's	4; 46 vs 4; 38	$P<0.0001$	****

References

1. Li H, Durbin R. Fast and accurate short read alignment with Burrows-Wheeler transform. Bioinformatics. 15 juill 2009;25(14):1754-60. doi:10.1093/bioinformatics/btp324 PubMed PMID: 19451168; PubMed Central PMCID: PMC2705234.

2. Fontaine F, Labalme A, Laurencin C, Theuriet J, Jacquier A, Lacoste N, et al. Homozygous COQ9 mutation: a new cause of potentially treatable hereditary spastic paraplegia. Eur J Hum Genet. 27 juin 2025. doi:10.1038/s41431-025-01895-w PubMed PMID: 40579432.

3. Richards S, Aziz N, Bale S, Bick D, Das S, Gastier-Foster J, et al. Standards and guidelines for the interpretation of sequence variants: a joint consensus recommendation of the American College of Medical Genetics and Genomics and the Association for Molecular Pathology. Genet Med. mai 2015;17(5):405-24. doi:10.1038/gim.2015.30 PubMed PMID: 25741868; PubMed Central PMCID: PMC4544753.

4. Schwarz JM, Cooper DN, Schuelke M, Seelow D. MutationTaster2: mutation prediction for the deep-sequencing age. Nat Methods. avr 2014;11(4):361-2. doi:10.1038/nmeth.2890 PubMed PMID: 24681721.

5. Sim NL, Kumar P, Hu J, Henikoff S, Schneider G, Ng PC. SIFT web server: predicting effects of amino acid substitutions on proteins. Nucleic Acids Res. juill 2012;40(Web Server issue):W452-457. doi:10.1093/nar/gks539 PubMed PMID: 22689647; PubMed Central PMCID: PMC3394338.

6. Adzhubei IA, Schmidt S, Peshkin L, Ramensky VE, Gerasimova A, Bork P, et al. A method and server for predicting damaging missense mutations. Nat Methods. avr 2010;7(4):248-9. doi:10.1038/nmeth0410-248

7. Ioannidis NM, Rothstein JH, Pejaver V, Middha S, McDonnell SK, Baheti S, et al. REVEL: An Ensemble Method for Predicting the Pathogenicity of Rare Missense Variants. The American Journal of Human Genetics. 6 oct 2016;99(4):877-85. doi:10.1016/j.ajhg.2016.08.016

8. Choi Y, Chan AP. PROVEAN web server: a tool to predict the functional effect of amino acid substitutions and indels. Bioinformatics. 15 août 2015;31(16):2745-7. doi:10.1093/bioinformatics/btv195 PubMed PMID: 25851949; PubMed Central PMCID: PMC4528627.

9. Rentzsch P, Witten D, Cooper GM, Shendure J, Kircher M. CADD: predicting the deleteriousness of variants throughout the human genome. Nucleic Acids Res. 8 janv 2019;47(D1):D886-94. doi:10.1093/nar/gky1016 PubMed PMID: 30371827; PubMed Central PMCID: PMC6323892.

10. Cheng J, Novati G, Pan J, Bycroft C, Žemgulytė A, Applebaum T, et al. Accurate proteome-wide missense variant effect prediction with AlphaMissense. Science. 22 sept 2023;381(6664):eadg7492. doi:10.1126/science.adg7492 PubMed PMID: 37733863.

11. de Sainte Agathe JM, Filser M, Isidor B, Besnard T, Gueguen P, Perrin A, et al. SpliceAI-visual: a free online tool to improve SpliceAI splicing variant interpretation. Hum

Genomics. 10 févr 2023;17(1):7. doi:10.1186/s40246-023-00451-1 PubMed PMID: 36765386; PubMed Central PMCID: PMC9912651.

12. Emsley P, Cowtan K. Coot: model-building tools for molecular graphics. Acta Crystallogr D Biol Crystallogr. déc 2004;60(Pt 12 Pt 1):2126-32. doi:10.1107/S0907444904019158 PubMed PMID: 15572765.

13. McNicholas S, Potterton E, Wilson KS, Noble MEM. Presenting your structures: the CCP4mg molecular-graphics software. Acta Crystallogr D Biol Crystallogr. avr 2011;67(Pt 4):386-94. doi:10.1107/S0907444911007281 PubMed PMID: 21460457; PubMed Central PMCID: PMC3069754.

14. Pellegrino C, Gubkina O, Schaefer M, Becq H, Ludwig A, Mukhtarov M, et al. Knocking down of the KCC2 in rat hippocampal neurons increases intracellular chloride concentration and compromises neuronal survival. The Journal of physiology. mai 2011;589(Pt 10):2475-96. doi:10.1113/jphysiol.2010.203703

15. Buerli T, Pellegrino C, Baer K, Lardi-Studler B, Chudotvorova I, Fritschy JMJM, et al. Efficient transfection of DNA or shRNA vectors into neurons using magnetofection. Nature protocols. janv 2007;2(12):3090-101. doi:10.1038/nprot.2007.445

16. Jarolimek W, Lewen A, Misgeld U. A furosemide-sensitive K+-Cl- cotransporter counteracts intracellular Cl- accumulation and depletion in cultured rat midbrain neurons. The Journal of neuroscience : the official journal of the Society for Neuroscience. juin 1999;19(12):4695-704.

17. Kelsch W, Hormuzdi S, Straube E, Lewen A, Monyer H, Misgeld U. Insulin-like growth factor 1 and a cytosolic tyrosine kinase activate chloride outward transport during maturation of hippocampal neurons. The Journal of neuroscience : the official journal of the Society for Neuroscience. nov 2001;21(21):8339-47.

18. Khirug S, Yamada J, Afzalov R, Voipio J, Khiroug L, Kaila K. GABAergic depolarization of the axon initial segment in cortical principal neurons is caused by the Na-K-2Cl cotransporter NKCC1. The Journal of neuroscience : the official journal of the Society for Neuroscience. avr 2008;28(18):4635-9.

19. Puskarjov M, Seja P, Heron SE, Williams TC, Ahmad F, Iona X, et al. A variant of KCC2 from patients with febrile seizures impairs neuronal Cl- extrusion and dendritic spine formation. EMBO reports. mars 2014;15(6):723-9. doi:10.1002/embr.201438749

20. Uvarov P, Fudo S, Karakus C, Golubtsov A, Rotondo F, Sukhanova T, et al. Uncovering novel KCC2 regulatory motifs through a comprehensive transposon-based mutant library. Front Mol Neurosci. 15 janv 2025;17. doi:10.3389/fnmol.2024.1505722

21. Dumon C, Diabira DD, Chudotvorova I, Bader F, Sahin S, Zhang J, et al. The adipocyte hormone leptin sets the emergence of hippocampal inhibition in mice. eLife. 2018;7(pii):1-19. doi:10.7554/eLife.36726

22. Hartmann AM, Wenz M, Mercado A, Störger C, Mount DB, Friauf E, et al. Differences in the large extracellular loop between the K(+)-Cl(-) cotransporters KCC2 and KCC4. The Journal of biological chemistry. juill 2010;285(31):23994-4002. doi:10.1074/jbc.M110.144063

23. Becker L, Hausmann J, Hartmann AM. Both chloride-binding sites are required for KCC2-mediated transport. J Biol Chem. 23 août 2023;299(10):105190. doi:10.1016/j.jbc.2023.105190 PubMed PMID: 37625593; PubMed Central PMCID: PMC10518353.

24. Payne JA. Functional characterization of the neuronal-specific K-Cl cotransporter: implications for [K+]o regulation. The American journal of physiology. nov 1997;273(5 Pt 1):C1516-25.

25. Hershfinkel M, Kandler K, Knoch ME, Dagan-Rabin M, Aras MA, Abramovitch-Dahan C, et al. Intracellular zinc inhibits KCC2 transporter activity. Nature neuroscience. juin 2009;12(6):725-7.

26. Hamze M, Brier C, Buhler E, Zhang J, Medina I, Porcher C. Regulation of Neuronal Chloride Homeostasis by Pro- and Mature Brain-Derived Neurotrophic Factor (BDNF) via KCC2 Cation–Chloride Cotransporters in Rat Cortical Neurons. International Journal of Molecular Sciences. janv 2024;25(11):11. doi:10.3390/ijms25116253

27. Lemonnier E, Villeneuve N, Sonie S, Serret S, Rosier A, Roue M, et al. Effects of bumetanide on neurobehavioral function in children and adolescents with autism spectrum disorders. Translational Psychiatry. 2017;7(3):e1056-9. doi:10.1038/tp.2017.10

28. Xie Y, Chang S, Zhao C, Wang F, Liu S, Wang J, et al. Structures and an activation mechanism of human potassium-chloride cotransporters. Sci Adv. déc 2020;6(50):eabc5883. doi:10.1126/sciadv.abc5883 PubMed PMID: 33310850; PubMed Central PMCID: PMC7732191.
